# Tumor Cell‐Derived CXCL2 Potentiates Neutrophil‐Mediated Antitumor Immunity by Inhibiting Cholesterol Biosynthesis in Hepatocellular Carcinoma

**DOI:** 10.1002/advs.202511436

**Published:** 2025-10-20

**Authors:** Xin Liu, Danli Yang, Qianqian Jiang, Meng Han, Zhao Zhou, Yukun Li, Yu Wu, Jingzhou Wang, Ting Zhang, Guohua Lou, Guochao Wei, Lin Wang, Fengmin Lu, Xiangmei Chen

**Affiliations:** ^1^ Department of Microbiology and Infectious Disease Research Center School of Basic Medical Sciences Peking University Health Science Center Beijing 100191 China; ^2^ Peking University Hepatology Institute Peking University People's Hospital Beijing 100044 China; ^3^ Department of Laboratory Medicine The First Affiliated Hospital of Zhengzhou University Zhengzhou 450052 China; ^4^ State Key Laboratory for Diagnosis and Treatment of Infectious Diseases National Clinical Research Centre for Infectious Diseases Collaborative Innovation Centre for Diagnosis and Treatment of Infectious Diseases The First Affiliated Hospital Zhejiang University School of Medicine Hangzhou Zhejiang Province 310003 China

**Keywords:** C‐X‐C motif chemokine ligand 2 (CXCL2), cholesterol metabolism, neutrophil, polarization

## Abstract

Hepatocellular carcinoma (HCC) is a major cause of cancer‐related death worldwide. Despite the proven efficacy of immunotherapy against malignancies, a large proportion of patients with HCC fail to benefit from these efficacious agents because of their overwhelmingly immunosuppressive microenvironment. Therefore, there is an urgent need to identify key genes and develop effective strategies for reshaping the HCC microenvironment. Here, a significant downregulation of C‐X‐C motif chemokine ligand 2 (CXCL2) in HCC is identified due to gene copy number loss, which correlates with poor prognosis and suboptimal responsiveness to immunotherapy. Subsequently, it is found that CXCL2 can not only recruit neutrophils as expected, but also induce their polarization toward the antitumor type to curb HCC progression. Mechanistically, differing from the prevailing notion that CXCL2 primarily functions extracellularly as a chemokine, it is demonstrated that intracellular CXCL2 can bind to Y‐Box Binding Protein 1 (YBX1) and prevent its nuclear translocation. Consequently, this reduces the transcription of sterol regulatory element binding transcription factor 2 (SREBF2) and suppresses cholesterol biosynthesis, thereby remodeling HCC microenvironment and impeding HCC development. In summary, this study highlights the unconventional role of CXCL2 in regulating neutrophil polarization and immune responses in HCC, positioning it as a potential therapeutic target for HCC.

## Graphical abstract

This study identifies the unconventional role of CXCL2 in regulating neutrophil polarization and immune responses in HCC. Unlike the common view that CXCL2 acts mainly as an extracellular chemokine, intracellular CXCL2 can interact with YBX1 and prevent its nuclear translocation. This reduces SREBF2 transcriptional expression and decreases cholesterol biosynthesis, thereby reshaping the HCC microenvironment and inhibiting HCC progression.

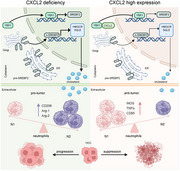

## 1 Introduction

Hepatocellular carcinoma (HCC) is one of the leading causes of cancer‐related mortality worldwide and is characterized by a complex interplay between genetic mutations, environmental factors, and immune system interactions.^[^ ^1^ ^]^ Despite advances in HCC therapeutics, the prognosis of HCC remains challenging due to its late‐stage diagnosis and early metastasis or recurrence. In recent years, many studies have illuminated the crucial role of immunotherapy in treating malignancies.^[^ ^2^, ^3^ ^]^ However, a majority of patients with HCC cannot benefit from such effective agents due to their overwhelmingly immunosuppressive microenvironment.^[^ ^4^ ^]^ Therefore, it is an urgent need to find effective ways to reshape the microenvironment.

Copy number variations (CNV), defined as an amplification or decrease in the number of DNA segments that are 1 kb or larger in the human genome, are frequent events in tumors.^[^ ^5^, ^6^ ^]^ Previous studies have shown that the duplication or deletion of CNV affects the expression of genes and cancer‐related biological processes. For example, Dong et al. observed a noteworthy association between CNV and differential gene expression in esophageal cancer, among which FAM60A was identified as a potential prognostic factor for overall survival.^[^ ^7^ ^]^ For HCC, researchers have identified the duplications of chromosome 1, 7, 8, and 20 and deletions of chromosome 4, 8, 13, and 17, mostly by comparative genomic hybridization analysis with genetic materials from tumor tissues.^[^ ^8^, ^9^, ^10^, ^11^ ^]^ Clifford et al. examined CNV in 386 patients with HCC and identified CNV in the ALDH7A1, C4orf29, and KNG1 genes, etc.^[^ ^12^ ^]^ Some of these genes have been proved to be related and targetable to the immune response and tumorigenesis.^[^ ^13^, ^14^, ^15^ ^]^ These results suggested that the use of high‐throughput technology to find the key genes with CNV is meaningful for the precision treatment of tumors. However, most existing studies have mainly focused on genes with copy number gains, overlooking potentially important genes with copy number deletions, which also have a great influence on cancer treatment. Therefore, exploring the functions of genes showing severe copy number losses during the HCC progression is necessary.

In the present study, we reanalyzed our previous array‐based comparative genomic hybridization (aCGH) and RNA expression profile results,^[^ ^16^, ^17^ ^]^ and found that the gene copy number of the C‐X‐C motif chemokine ligand 2 (CXCL2) was frequently deleted, resulting in its low expression in HCC tissues. Patients with low CXCL2 levels had poorer clinical outcomes. These findings are paradoxical to previous studies reporting that high CXCL2 levels in tumors are prone to shaping the immunosuppressive microenvironment by recruiting neutrophils.^[^ ^18^, ^19^ ^]^ However, a minority of studies have demonstrated that CXCL2 overexpression could inhibit HCC proliferation without detailed exploration.^[^ ^20^, ^21^ ^]^ This dichotomy in CXCL2 function highlights the need for a deeper understanding of its mechanisms in HCC progression and potentially, the impact on therapy. Our subsequent in vitro and in vivo experiments revealed that overexpression of CXCL2 promoted neutrophil polarization toward the antitumor phenotype (N1), thereby inhibiting HCC progression. Mechanistically, CXCL2 interacts with Y‐Box Binding Protein 1 (YBX1) and prevents its nuclear translocation to reduce sterol regulatory element binding transcription factor 2 (SREBF2) transcription, leading to decreased cholesterol synthesis and a remodeled antitumor immune microenvironment that retards HCC progression.

## 2 Results

### 2.1 CXCL2 is Down‐Regulated in HCC Tissues and Predicts Poor Prognosis

**Figure 1:**
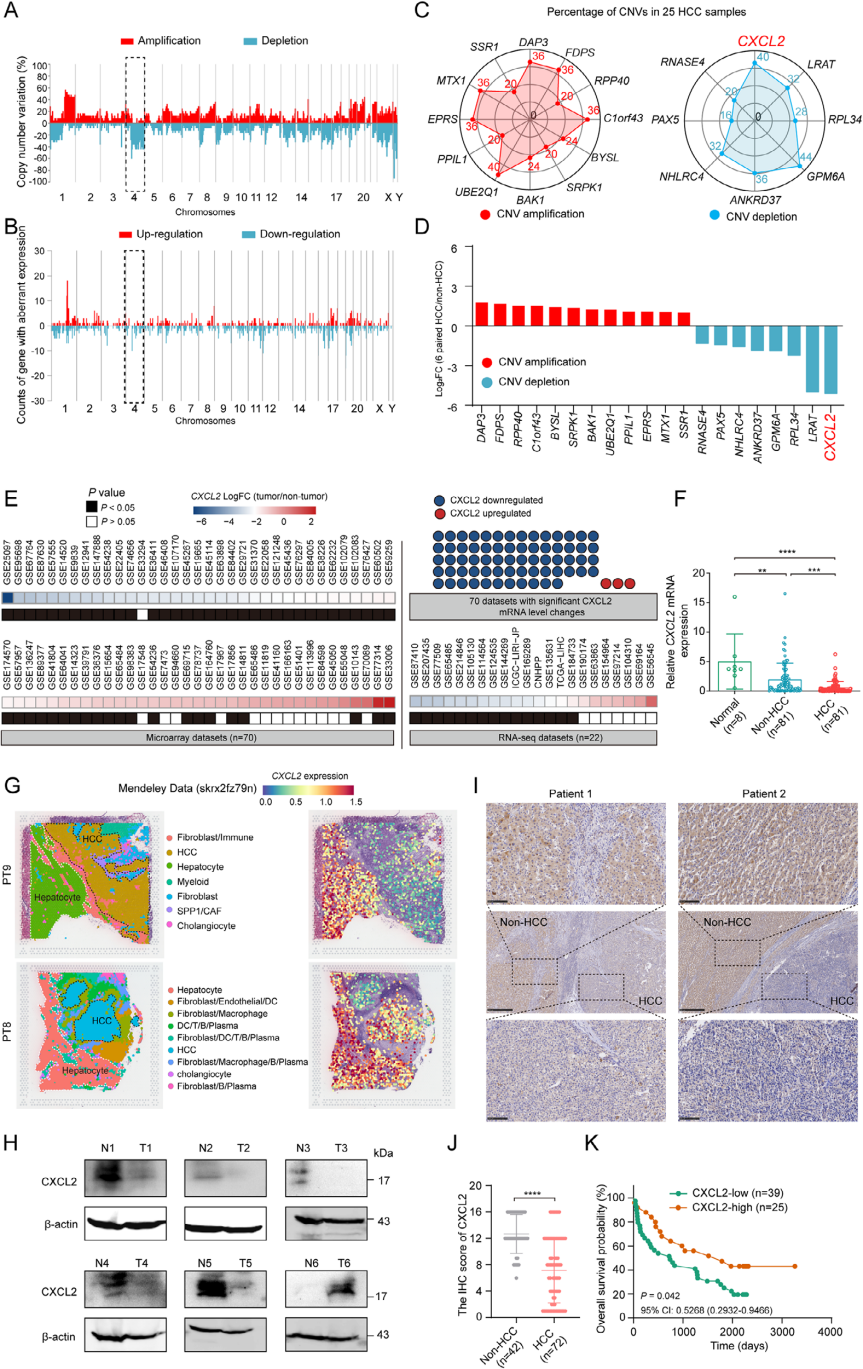
CXCL2 was downregulated in HCC tissues and predicted poor prognosis. A) The copy number variation (CNV) percentage of chromosomes using the array comparative genome hybridization chip of 25 paired HCC and adjacent non‐tumor tissues. B) The counts of genes with aberrant expression (Fold Change ≥2) in chromosomes using the transcriptome profile of 6 paired HCC and adjacent non‐tumor tissues. C) The CNV percentage of 20 candidate genes in 25 HCC tissues. D) The expression status of 20 candidate genes in 6 paired HCC and adjacent non‐tumor tissues. E) The expression profile of CXCL2 mRNA in HCC from the IHGA database. F) The RT‐qPCR analysis of CXCL2 mRNA in 81 paired HCC tissues and adjacent non‐tumor tissues as well as 8 normal liver tissues. G) The expression feature of CXCL2 mRNA from HCC spatial transcriptomics dataset from Mendeley database. H) The protein expression level of CXCL2 in 6 paired HCC and adjacent non‐tumor tissues by western blot. I) The representative immunohistochemistry (IHC) image of adjacent non‐tumor tissues and HCC tissues stained with CXCL2 antibody. J) The IHC score of CXCL2 was compared in adjacent non‐tumor tissues and HCC tissues. K) Kaplan‐Meier analysis of survival of patients with HCC classified by CXCL2 expression. Data are presented as the mean ± SD. ^**^ *P* < 0.01, ^***^ *P* < 0.001, ^****^ *P* < 0.0001, *P* value was calculated by one‐way ANOVA (F), Mann‐Whitney U test (J), log rank test (K).

To identify the key genes associated with CNVs that contributed to HCC progression, we reanalyzed our previous aCGH data on 25 paired adjacent non‐tumor tissues and HCC tissues, in conjunction with a transcriptome profiling in 6 pairs among the above specimens.^[^ ^16^, ^17^ ^]^ The results showed that the copy number and expression of genes in chromosome 4 were significantly depleted and downregulated, respectively, which was consistent with prior studies (**Figure** **1**A,B).^[^ ^11^ ^]^ Next, based on a Pearson analysis of CNVs and gene expression, we identified 20 candidate genes (12 showed CNV amplification and 8 showed CNV depletion). These genes had the absolute correlation coefficient of 1, meaning that their CNVs were perfectly correlated with their gene expression. Among them, the frequent CNV depletion (40%) and significantly low expression of CXCL2 piqued our interest (Figure 1C,D) because many studies have suggested that CXCL2 could attract neutrophils and reshape an immunosuppressive microenvironment, thereby facilitating HCC immune escape.^[^ ^18^, ^19^ ^]^ Only a minority of publications proposed that high CXCL2 expression could inhibit the malignant behavior of HCC.^[^ ^20^, ^21^ ^]^ Concordantly, the CXCL2 gene also had omnibus deletion in HCC patients from the TCGA‐LIHC dataset (Figure S1A, Supporting Information). Furthermore, we retrieved the bulk RNA sequencing data of HCC from the Integrative HCC Gene Analysis (IHGA) database.^[^ ^22^ ^]^ The results demonstrated that the expression levels of CXCL2 were significantly lower in HCC tissues than in adjacent tissues, which was consistent with our real‐time quantitative PCR (RT‐qPCR) analysis of HCC tissues (Figure 1E,F). Additionally, spatial transcriptomics data obtained from Mendeley Data and HCCDB database indicated a marked reduction in CXCL2 expression in HCC cells (Figure 1G; Figure S1B–H, Supporting Information). Furthermore, our clinical samples revealed a substantial decrease in CXCL2 protein levels in HCC tissues than those in adjacent non‐tumor tissues (Figure 1H–J). Similar results were obtained in the mouse models of spontaneous HCC (Figure S1I, Supporting Information). Survival analysis indicated that patients with high CXCL2 expression had more favorable clinical outcomes (Figure 1K; Figure S1J,K, Supporting Information). These findings imply that CXCL2 is downregulated in HCC due to a high frequency of gene copy number deletions and might serve as a potential tumor suppressor in HCC.

### 2.2 CXCL2 Promotes Antitumor Immunity in a Neutrophil‐Dependent Manner

**Figure 2:**
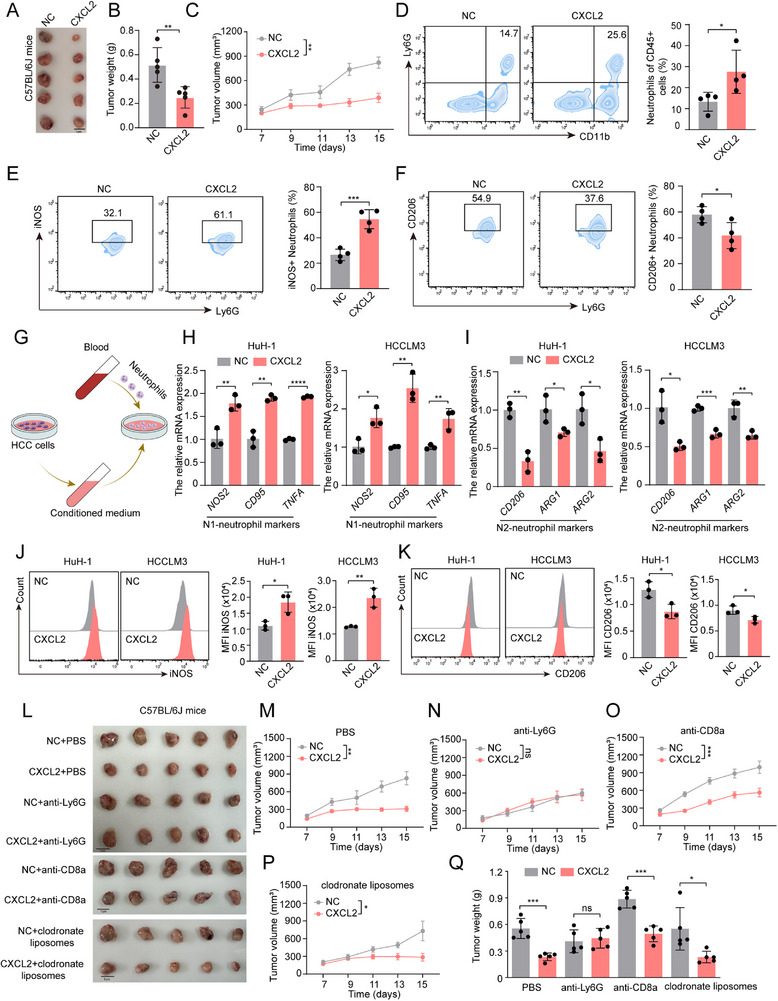
CXCL2 suppressed HCC progression through promoting neutrophil polarization toward antitumor phenotype. A) The image of subcutaneous tumor in C57BL/6J mice inoculated with negative control (NC) or CXCL2 overexpression (CXCL2) Hepa 1–6 cells. B) The comparison of tumor weight in C57BL/6J mice inoculated with NC or CXCL2 Hepa 1–6 cells (n = 5). C) The tumor growth curve in C57BL/6J mice inoculated with NC or CXCL2 Hepa 1–6 cells (n = 5). D–F) FCM analysis of the proportion of tumor‐infiltrating neutrophils, iNOS^+^ neutrophils and CD206^+^ neutrophils (n = 4). G) The illustration of the co‐culture experiment. H,I) The relative mRNA expression of N1‐neutrophil markers and N2‐neutrophil markers in neutrophils co‐cultured with the conditional medium from NC or CXCL2 overexpression HCC cells for 12h, respectively (n = 3). J,K) The FCM analysis of iNOS and CD206 in neutrophils co‐cultured with the conditional medium from NC or CXCL2 overexpression HCC cells for 12h, respectively (n = 3). L) The image of subcutaneous tumor in C57BL/6J mice inoculated with NC or CXCL2 overexpression Hepa 1–6 cells treated with PBS, anti‐Ly6G, anti‐CD8, and clodrondate liposomes, respectively. M–P) The tumor growth curve in C57BL/6J mice inoculated with NC or CXCL2 overexpression Hepa 1–6 cells and treated with PBS, anti‐Ly6G, anti‐CD8 and clodrondate liposomes, respectively (n = 5). Q) The comparison of tumor weight in C57BL/6J mice inoculated with NC or CXCL2 overexpression treated with PBS, anti‐Ly6G, anti‐CD8 and clodrondate liposomes, respectively (n = 5). Data are presented as the mean ± SD (B, D, E, F, H, I, J, K and Q) or mean ± SEM (C, M, N, O and P). ^*^ *P* < 0.05, ^**^ *P* < 0.01, ^***^ *P* < 0.001, ^****^ *P* < 0.0001, ns, no significance. *P* value was calculated by Student's *t* test (B, D, E, F, H, I, J, K and Q) and two‐way ANOVA (C, M, N, O and P).

On the basis of these findings, we investigated the role of CXCL2 in HCC. Next, we conducted in vitro and in vivo experiments with the stable CXCL2 overexpression HCC cells (Figure S2A, Supporting Information). CCK‐8 and colony formation assays revealed that CXCL2 overexpression had no significant impact on the proliferative capacity of HCC cells (Figure S2B,C, Supporting Information). Interestingly, subcutaneous tumors in C57BL/6J mice revealed that tumors in the CXCL2 overexpression group grew at a slower rate and were smaller in volume than those in the negative control (NC) group (**Figure** **2**A–C). Flow cytometry (FCM) results showed that the CXCL2 overexpression group had a higher number of neutrophils, consistent with previous studies (Figure 2D).^[^ ^18^, ^23^ ^]^ Notably, we found that neutrophils in the CXCL2 overexpression group were predominantly positive for inducible nitric oxide synthase (iNOS) but not CD206, indicating that these neutrophils were polarized to an antitumor phenotype (N1) under CXCL2 overexpression (Figure 2E,F). The proportion and function of other types of immune cells showed no significant changes (Figure S3A–H, Supporting Information). These results suggest that CXCL2 promotes neutrophil infiltration and drives their polarization toward an antitumor phenotype, thereby inhibiting HCC progression.

To better understand the role of CXCL2 in neutrophils, we reanalyzed a single‐cell RNA sequencing (scRNA‐seq) dataset from HCC patients (GSE242889).^[^ ^24^ ^]^ Based on CXCL2 mRNA expression in malignant cells, these patients were divided into CXCL2 high (n = 3) and low (n = 2) groups (Figure S4A–C, Supporting Information). We observed that neutrophils in the CXCL2‐high group exhibited a significantly higher proportion of the antitumor N1 phenotype (Figure S4D–G, Supporting Information). Similar results were further validated in independent HCC bulk RNA‐seq data from TCGA and GEO databases, where elevated CXCL2 expression consistently correlated with N1‐polarized neutrophils (Figure S4H,I, Supporting Information). To further confirm these findings, we isolated primary neutrophils from human peripheral blood and examined the effects of CXCL2 on neutrophil function in vitro (Figure 2G). RT‐qPCR results demonstrated that neutrophils exposed to conditioned medium from CXCL2 overexpressing HCC cells showed significantly higher expression of iNOS and other N1 markers, but lower expression of CD206 and other N2 markers, compared to neutrophils exposed to the conditioned medium from the NC group (Figure 2H,I). Similar results were obtained from FCM analysis (Figure 2J,K). In addition, the apoptosis rate of neutrophils was comparable between the NC and CXCL2 overexpression groups (Figure S5A,B, Supporting Information). These results indicated that CXCL2 could promote neutrophils polarization toward an antitumor phenotype.

To further elucidate whether CXCL2 relies on neutrophils to exert its antitumor effect, we selectively depleted of neutrophils, CD8^+^ T cells, and macrophages in C57BL/6J mice bearing HCC cells, respectively (Figure S5C, Supporting Information). Immunohistochemistry (IHC) validated the efficacy of each depletion protocol (Figure S5D–F, Supporting Information). Consistent with previously reported,^[^ ^25^, ^26^, ^27^ ^]^ no significant change in tumor volume was observed after the depletion of neutrophils and macrophages, while a slight increase in tumor volume was noted following the clearance of CD8^+^ T cells (Figure 2L). Strikingly, neutrophil depletion abolished the antitumor activity of CXCL2, with no significant differences observed in tumor growth between CXCL2 overexpression and control groups (Figure 2L–N, Q). In contrast, CXCL2 overexpression retained its tumor‐suppressive effects in mice depleted of CD8^+^ T cells or macrophages (Figure 2L, O–Q). Although it has been reported that clodronate liposomes can impair neutrophil function in the genetic models of mononuclear phagocytes (MoPh) deficiency,^[^ ^28^ ^]^ we found that following clodronate liposomes treatment, CXCL2 overexpression could still induce the antitumor function of neutrophils (Figure S5G, Supporting Information). Taken together, these findings suggest that CXCL2 represses HCC progression via neutrophil‐mediated antitumor immunity.

### 2.3 Intracellular CXCL2 Inhibits HCC Progression Through Promoting Neutrophils Polarization Toward Antitumor Phenotype

**Figure 3:**
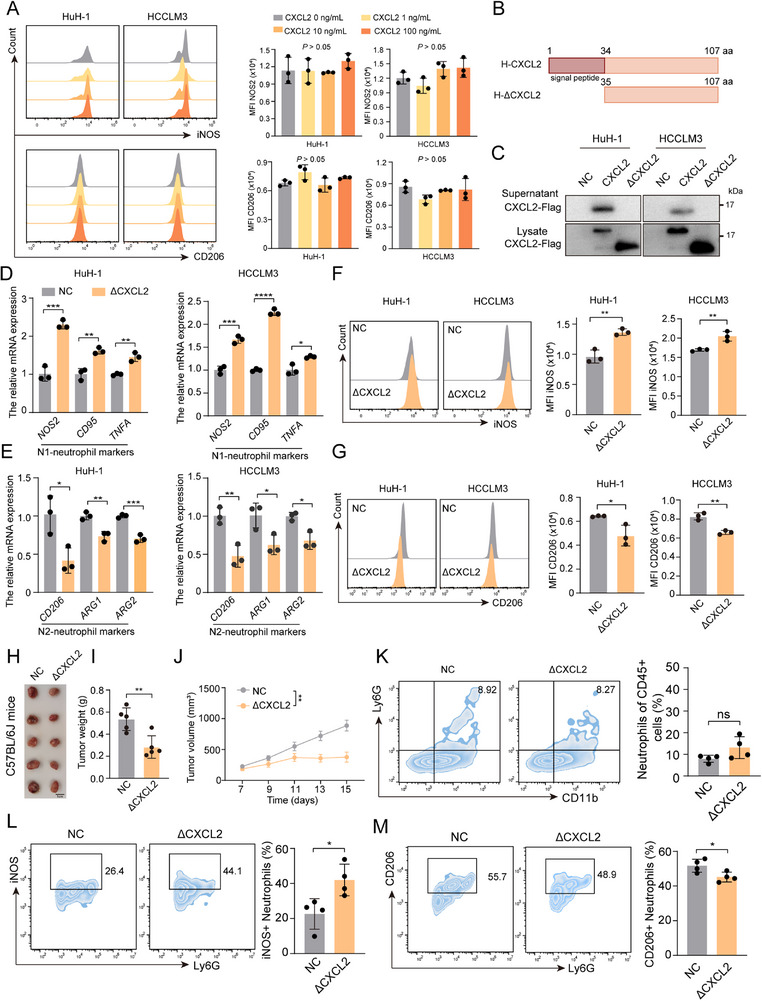
Intracellular CXCL2 inhibited HCC progression through promoting neutrophils polarization toward antitumor phenotype. A) The FCM analysis of iNOS and CD206 in neutrophil co‐cultured with the conditional medium of HCC cells supplemented with 0, 1, 10, or 100 ng mL^−1^ recombinant exogenous CXCL2 protein for 12h, respectively (n = 3). B) The schematic representation of the full‐length CXCL2 and truncated mutant with signal peptide deletion (ΔCXCL2). C) The supernatants and cell lysates from HCC cells with Flag‐tagged CXCL2 or ΔCXCL2 overexpressed were determined by western blot using an anti‐Flag antibody. D,E) The relative mRNA expression of N1‐neutrophil markers and N2‐neutrophil markers in neutrophils co‐cultured with the conditional medium from NC or ΔCXCL2 overexpression HCC cells for 12h, respectively (n = 3). F,G) The FCM analysis of iNOS and CD206 in neutrophils co‐cultured with the conditional medium from NC or ΔCXCL2 overexpression HCC cells for 12h, respectively (n = 3). H) The image of subcutaneous tumor in C57BL/6J mice inoculated with NC or ΔCXCL2 overexpression Hepa 1–6 cells. I) The comparison of tumor weight in C57BL/6J mice inoculated with NC or ΔCXCL2 overexpression Hepa 1–6 cells (n = 5). J) The tumor growth curve in C57BL/6J mice inoculated with NC or ΔCXCL2 overexpression Hepa 1–6 cells (n = 5). K–M) The FCM analysis of the proportion of tumor‐infiltrating neutrophils, iNOS^+^ neutrophils and CD206^+^ neutrophils (n = 4). Data are presented as the mean ± SD (A, D, E, F, G, I, K, L and M) or mean ± SEM (J). ^*^ *P* < 0.05, ^**^ *P* < 0.01, ^***^ *P* < 0.001, ns, no significance. *P* value was calculated by one‐way ANOVA (A), Student's t test (D, E, F, G, I, K, L and M) and two‐way ANOVA (J).

Building on emerging evidence that certain secreted proteins exhibit distinct intra‐ and extracellular functions, we hypothesized that CXCL2, a chemokine classically associated with neutrophil recruitment, might similarly display dual functionality.^[^ ^29^ ^]^ To evaluate this, we first investigated the extracellular role of CXCL2 by treating HCC cells with recombinant CXCL2 protein. Intriguingly, although exogenous CXCL2 had no statistically significant effect on neutrophil polarization, it markedly enhanced HCC cell proliferation in vitro (**Figure** **3**A; Figure S6A,B, Supporting Information). Subsequently, we constructed a CXCL2 construct lacking the signal peptide (named as ΔCXCL2), which could retain CXCL2 intracellularly confirmed by western blot (Figure 3B,C; Figure S6C,D, Supporting Information). Surprisingly, we found that ΔCXCL2 markedly inhibited HCC cell proliferation (Figure S6E–G, Supporting Information). Furthermore, RT‐qPCR analysis and FCM experiments also revealed that neutrophils co‐cultured with HCC cells expressing ΔCXCL2 exhibited higher N1 phenotype markers and a lower marker of N2 phenotype compared to those co‐cultured with control HCC cells, with no obvious alterations of apoptosis rate (Figure 3D–G; Figure S6H,I, Supporting Information). These observations were validated further by in vivo experiments, which demonstrated that tumors expressing ΔCXCL2 grew more slowly and had a smaller weight compared to the control group (Figure 3H–J). The FCM analysis showed that, although no significant difference in neutrophils infiltration between the two groups due to the absence of signal peptide, the neutrophils in the ΔCXCL2 group exhibited significantly enhanced antitumor (N1) functional characteristics (Figure 3K–M). Collectively, these findings suggest that intracellular CXCL2 promotes antitumor immune functions of neutrophils.

### 2.4 CXCL2 Inhibits Cholesterol Biosynthesis in HCC

**Figure 4:**
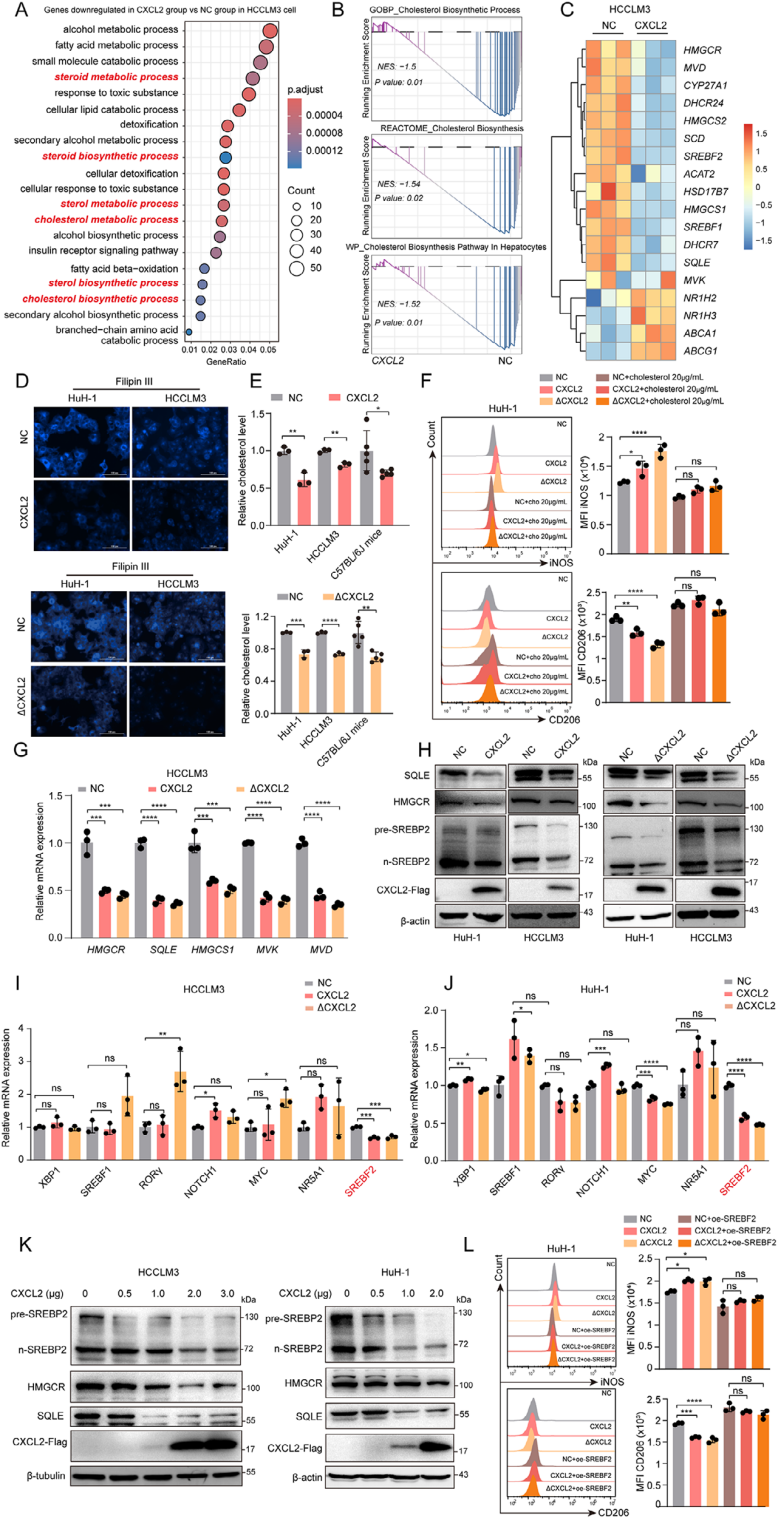
CXCL2 inhibited cholesterol biosynthesis in HCC. A) The GO analysis of genes downregulated in CXCL2 overexpression group vs NC group in HCCLM3 cells. B) The GSEA result of gene signature of cholesterol biosynthesis pathway in NC and CXCL2 overexpression groups of HCCLM3 cells. C) The heatmap of cholesterol biosynthesis related genes expression in NC and CXCL2 overexpression group of HCCLM3 cells. D,E) The representative image of Filipin III staining of cholesterol and the relative cholesterol measurement in NC and CXCL2/ΔCXCL2 overexpression HCC cells. F) The FCM analysis of iNOS and CD206 in neutrophils co‐cultured with the conditional medium from NC and CXCL2/ΔCXCL2 overexpression HCC cells plus with 20 µg mL^−1^ cholesterol for 18 h, respectively (n = 3). G) The relative mRNA expression of cholesterol biosynthesis related genes in NC and CXCL2/ΔCXCL2 overexpression HCCLM3 cells (n = 3). H) The relative protein expression of cholesterol biosynthesis related genes in NC and CXCL2/ΔCXCL2 overexpression HCC cells were detected by western blot. I,J) The relative mRNA expression of key transcription factors for cholesterol biosynthesis in NC and CXCL2/ΔCXCL2 overexpression HCCLM3 and HuH‐1 cells (n = 3). K) The relative protein expression of cholesterol biosynthesis related genes in HCC cells transfected with different amount of CXCL2 overexpression plasmid was detected by western blot. L) The FCM analysis of iNOS and CD206 in neutrophil co‐cultured with the conditional medium from NC and CXCL2/ΔCXCL2 overexpression HCC cells with SREBF2 overexpression, respectively (n = 3). Data are presented as the mean ± SD. ^*^ *P* < 0.05, ^**^ *P* < 0.01, ^***^ *P* < 0.001, ^****^ *P* < 0.0001, ns, no significance. *P* value was calculated by Student's *t* test (E) and one‐way ANOVA (F, G, I, J and L).

To determine the underlying mechanism of CXCL2 in antitumor immunity, RNA‐seq was performed on CXCL2 overexpression and NC groups of HCCLM3 cells. The results of gene ontolgy (GO) of the differentially expressed gene profile showed significant enrichment in the cholesterol metabolism pathway (**Figure** **4**A). Gene Set Enrichment Analysis (GSEA) further revealed that compared to the CXCL2 overexpression group, the cholesterol biosynthesis pathway was significantly activated in the NC group, which was consistent with the analysis of TCGA and GEO databases (Figure 4B; Figure S7A, Supporting Information). Moreover, the RNA‐seq data indicated low expression levels of key molecules in the cholesterol biosynthetic pathway, such as SREBF2, HMGCR, SQLE, and HMGCS1 (Figure 4C). Subsequently, to directly visualize and quantitatively assess the effect of CXCL2 on cholesterol levels, we performed Filipin III and cholesterol content assays. The results clearly showed a marked reduction in cholesterol levels in CXCL2/ΔCXCL2 overexpression HCC cells compared to the control cells (Figure 4D,E; Figure S7B,C, Supporting Information). FCM assays further confirmed that the addition of cholesterol to the culture medium inhibited the CXCL2‐induced N1 phenotype (Figure 4F). This observation highlights CXCL2's role in modulating neutrophil polarization by suppressing cholesterol synthesis.

Next, we investigated how CXCL2 regulates the cholesterol levels. Subsequent RT‐qPCR and western blot results showed decreased expression levels of key genes in cholesterol biosynthesis pathways (Figure 4G,H; Figure S7D–F, Supporting Information). Given the widespread downregulation of cholesterol biosynthesis‐related molecules, we hypothesized that CXCL2 affects cholesterol synthesis by targeting key transcription factors (TFs). We examined the expression of several important TFs involved in the cholesterol synthesis pathway including SREBF2, SREBF1, NR5A1, NOTCH1, XBP1, RORγ, and MYC.^[^ ^30^, ^31^ ^]^ The results showed that compared with the other TFs, the expression of SREBF2 was significantly reduced in both of the CXCL2 and ΔCXCL2 overexpression groups at the mRNA level (Figure 4I,J). Moreover, western blot also showed that SREBP2 was downregulated in CXCL2/ΔCXCL2 overexpression group (Figure 4H; Figure S7F, Supporting Information). In line with this, a significant negative correlation between CXCL2 and SREBF2 transcription was observed public HCC data from GEO database (Figure S7G, Supporting Information). More importantly, when varying amounts of CXCL2‐expressing plasmids were transfected into HCC cells, we found a corresponding decrease in the expression levels of SREBP2 and other key proteins involved in cholesterol synthesis as the intracellular CXCL2 protein increased within the cells (Figure 4K). Moreover, FCM assay revealed that the CXCL2‐induced N1 phenotype was suppressed by SREBF2 overexpression (Figure 4L). These findings suggest that CXCL2 influences the antitumor function of neutrophils through regulating SREBF2‐mediated cholesterol biosynthesis in HCC.

### 2.5 CXCL2 Reduces SREBF2 Transcription by Interacting with YBX1 and Retarding its Nuclear Translocation

**Figure 5:**
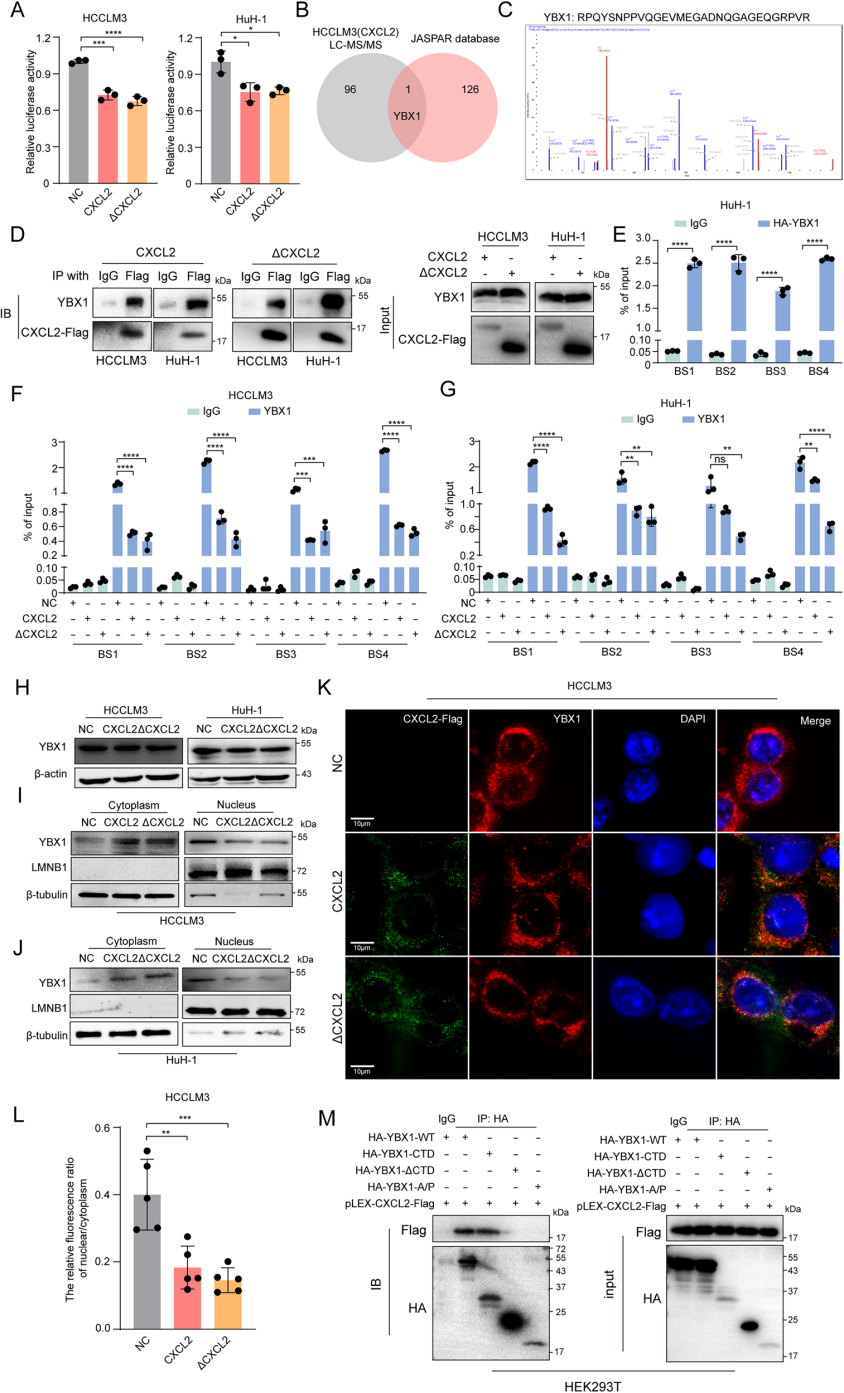
CXCL2 reduces SREBF2 transcription by interacting with YBX1 and retarding its nuclear translocation. A) The dual‐luciferase activity was measured after 48h transfected with SREBF2 promoter luciferase reporter plasmids and Actin‐Renilla vectors into HCCLM3 or HuH‐1 cells with CXCL2/ΔCXCL2 overexpression (n = 3). B) The venn diagram showed the shared genes between proteins interacted with CXCL2 by LC‐MS/MS and putative transcription factors of SREBF2 by JASPAR database. C) Identification of YBX1 by LC‐MS/MS. D) Co‐IP assay was performed in CXCL2/ΔCXCL2 overexpression HCC cells using anti‐Flag antibody. E) ChIP‐qPCR assay was performed using IgG or anti‐HA antibody in HuH‐1 cells transfected with pcDNA3.1‐HA‐YBX1 plasmid for 48h (n = 3). F,G) ChIP‐qPCR assay was performed using IgG or anti‐YBX1 antibody in CXCL2/ΔCXCL2 overexpression HCCLM3 and HuH‐1 cells (n = 3). H) The expression of YBX1 in CXCL2/ΔCXCL2 overexpression was explored by western blot. I,J) The expression of YBX1 in cytoplasm and nucleus was explored by western blot in CXCL2/ΔCXCL2 overexpression HCCLM3 and HuH‐1 cells. K) The co‐localization of CXCL2 with YBX1 detected by confocal microscope. L) The relative fluorescence ratio of nuclear/cytoplasm of YBX1 in NC/CXCL2/ΔCXCL2 overexpression in HCCLM3 cell (n = 5). M) HEK293T cells were co‐transfected with pLEX‐CXCL2‐Flag plasmid with YBX1 truncated mutants for 48 h. Co‐IP assay was performed using IgG or anti‐HA antibody. Data are presented as the mean ± SD. ^*^ *P* < 0.05, ^**^ *P* < 0.01, ^***^ *P* < 0.001, ^****^ *P* < 0.0001, ns, no significance. *P* value was calculated by one‐way ANOVA (A, F, G and L), Student's *t* test (E).

To further elucidate the molecular mechanism by which CXCL2 mediates the reduction in SREBF2 transcription, we initiated a series of experiments to understand the direct and indirect effects of CXCL2 on SREBF2 expression. The dual‐luciferase reporter assay and RNA stability experiments showed that either CXCL2 or ΔCXCL2 overexpression would significantly reduce the promoter activity of SREBF2 but not its RNA stability, suggesting that CXCL2 affected SREBF2 transcription rather than mRNA degradation (**Figure** **5**A; Figure S8A,B, Supporting Information). Considering that CXCL2 is not localized to the nucleus, we hypothesized that CXCL2 might inhibit SREBF2 transcription by influencing TFs that bind to the SREBF2 promoter. To verify this, LC‐MS/MS was conducted accompanied with the JASPAR public database to identify the potential target molecules. Delightedly, transcription factor YBX1 was obtained via the selection criteria as unique peptides ≥2 and Abundance Ratio: (IP) / (IgG) ≥2 in LC‐MS/MS analysis and Relative score> 0.99 in the JASPAR database (Figure 5B,C; Tables S1 and S2, Supporting Information). Then co‐immunoprecipitation (Co‐IP) assay was employed to confirm the interaction between CXCL2 and YBX1 (Figure 5D). Previous studies have reported that YBX1 plays an important role in oncogene expression and tumor progression by directly binding to RNA or DNA.^[^ ^32^, ^33^ ^]^ Although our RIP assay failed to show the binding of YBX1 to SREBF2 mRNA (Figure S8C, Supporting Information), ChIP‐qPCR experiments demonstrated that YBX1 was able to bind to the promoter region of SREBF2 gene (Figure 5E; Figure S8D, Supporting Information). Importantly, compared with the control group, CXCL2 overexpression significantly weakened YBX1's binding affinity to the SREBF2 promoter (Figure 5F,G). To further elucidate the role of YBX1 in mediating CXCL2's regulation of SREBF2, we investigated whether CXCL2 influenced YBX1 expression and subcellular localization. While CXCL2 overexpression did not markedly alter total YBX1 protein levels, it significantly decreased YBX1 nuclear localization, which was also confirmed by confocal microscopy (Figure 5H–L). In addition, the co‐IP results showed that the CTD domain of YBX1 was crucial for its interaction with CXCL2 (Figure 5M; Figure S8E,F, Supporting Information). In summary, our findings showed that CXCL2 inhibits SREBF2 transcription by interacting with YBX1's CTD domain, preventing YBX1 nuclear translocation and reducing its binding to the SREBF2 promoter.

### 2.6 CXCL2 Enhances the Treatment Efficacy of PD‐1 Antibody on HCC

**Figure 6:**
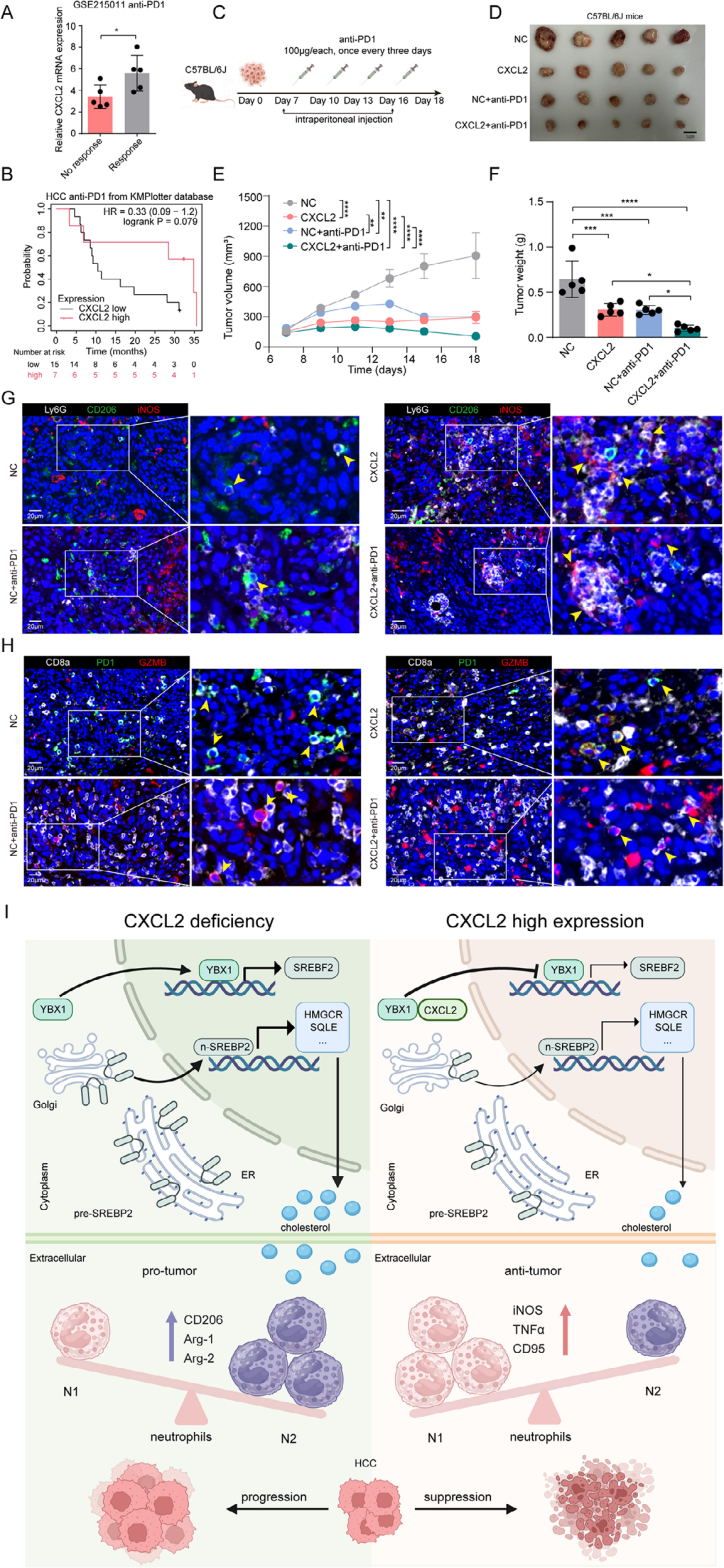
CXCL2 enhanced anti‐PD‐1 efficacy in HCC. A) The CXCL2 mRNA expression in HCC patients who were responsive or non‐responsive to anti‐PD‐1 treatment in GSE215011 dataset (n = 5). B) Survival analysis of HCC patients treated with anti‐PD1 based on CXCL2 mRNA expression from KMPlotter database. C) Treatment schedule of anti‐PD‐1 in the C57BL/6J mice inoculated with NC or CXCL2 overexpression Hepa 1–6 cells. D) The image of subcutaneous tumor in C57BL/6J mice. E) The tumor growth curve in C57BL/6J mice (n = 5). F) The comparison of tumor weight in C57BL/6J mice (n = 5). G,H) mIHC analysis displays the staining of Ly6G, CD206, iNOS and CD8A, PD1 and GZMB. I) The schematic diagram showing that CXCL2 overexpression promotes neutrophil polarization toward the antitumor phenotype (N1) to inhibit HCC progression. Mechanistically, CXCL2 interacts with YBX1 and prevents its nuclear translocation to reduce SREBF2 transcription, leading to decreased cholesterol synthesis and a remodeled antitumor immune microenvironment to retard HCC progression (Created in BioRender. Zhou, Z. (2025) https://BioRender.com/9qtkgp9). Data are presented as the mean ± SD (A and F) and mean ± SEM (E). ^*^ *P* < 0.05, ^**^ *P* < 0.01, ^***^ *P* < 0.001, ^****^ *P* < 0.0001. *P* value was calculated by, Student's *t* test (A), log‐rank test (B), two‐way ANOVA (E) and one‐way ANOVA (F).

These findings drove us to investigate whether CXCL2 overexpression improved the therapeutic efficacy of anti‐PD‐1 in patients with HCC. First, through analysis of the public HCC dataset from the TCGA and GEO databases, we observed that patients with high CXCL2 expression exhibited a higher immunophenoscore (IPS), which indicated enhanced suitability for immunotherapy, and were more prone to benefit from anti‐PD‐1 treatment (**Figure** **6**A; Figure S9A, Supporting Information). Prognostic analysis further revealed that immunotherapy outcomes might be more favorable in HCC patients with CXCL2 high expression (Figure 6B). Besides, we also observed a similar phenomenon in melanoma and glioblastoma (GBM) tumors (Figure S9B–I, Supporting Information). Subsequently, to corroborate these findings, subcutaneous tumor xenograft experiments were conducted in C57BL/6J mice (Figure 6C). The results demonstrated that the combined overexpression of CXCL2 with PD‐1 blockade markedly attenuated tumor growth and weight (Figure 6D–F). The mIHC analysis showed much more iNOS^+^ neutrophil and GZMB^+^ CD8^+^ T cell infiltration and fewer PD1^+^ CD8^+^ T cells in the tumor tissues of those mice treated with combination therapy (Figure 6G,H). These findings highlight the pivotal role of CXCL2 in modulating the tumor immune microenvironment and the efficacy of immunotherapy in HCC.

## 3 Discussion

A considerable number of studies have investigated the conversion of cold tumors into hot tumors to enhance the efficacy of immunotherapy, such as immune checkpoint inhibitors targeting PD‐1 and CTLA4.^[^ ^34^ ^]^ To achieve this transition, current efforts are primarily focused on enhancing immune cell infiltration and cytotoxic functions.^[^ ^35^, ^36^ ^]^ In recent years, neutrophils have garnered increasing attention, with many studies emphasizing their pivotal role in immune regulation.^[^ ^37^, ^38^ ^]^ Although neutrophils can exert antitumor effects under certain circumstances, they are frequently polarized into a pro‐tumorigenic type and facilitate tumor immune escape due to the immunosuppressive nature of tumors.^[^ ^39^ ^]^ Therefore, it is of utmost significance to identify the key molecules capable of modulating neutrophil polarization. In this study, we found that CXCL2 was significantly downregulated in HCC owing to its gene copy number deletion. CXCL2 overexpression enhances antitumor immunity by promoting neutrophil recruitment and functional polarization toward an antitumor phenotype.

In contrast to the prevailing view that CXCL2 mainly acts as an extracellular chemokine and promotes immune escape by recruiting neutrophils to tumors, our mechanistic analysis revealed a novel insight that intracellular CXCL2 reduced cholesterol biosynthesis by interacting with YBX1 and retarding its nuclear translocation for SREBF2 transcription (Figure 6I). Meaningfully, CXCL2 overexpression combined with anti‐PD‐1 therapy exhibited prominent tumor inhibition in mouse models. These findings highlight the unconventional role of CXCL2 in orchestrating neutrophil‐mediated antitumor immunity through its regulatory interplay with cholesterol biosynthesis pathways in HCC.

Functional duality of proteins, dictated by their subcellular localization and interaction networks, is a hallmark of cancer biology. For example, accumulating evidence has shown that the binding of extracellular fibroblast growth factor 2 (FGF2) to cell‐surface receptors can trigger a signaling cascade that spurs cell proliferation, migration, and angiogenesis.^[^ ^40^, ^41^ ^]^ In contrast, another study demonstrated that intracellular FGF2 promotes the differentiation of breast cancer cells to inhibit metastasis.^[^ ^42^ ^]^ Here, in contrast to most previous studies,^[^ ^43^ ^]^ we discovered that intracellular CXCL2 inhibits the proliferation of tumor cells and promotes the polarization of neutrophils toward an antitumor phenotype by suppressing cholesterol biosynthesis. More importantly, we observed that with an increasing quantity of transfected CXCL2 overexpression plasmids, there was an accumulation of CXCL2 protein within the cells. Concurrently, the expression of the pivotal molecules involved in the modulation cholesterol biosynthesis also underwent a gradual downregulation. Therefore, we postulated that once CXCL2 managed to accumulate intracellularly, it was likely to manifest antitumor efficacy. Moreover, based on experimental findings and a literature review, we found that CXCL2 is typically secreted upon synthesis and barely accumulated substantially within cells. Consequently, previous studies predominantly focused on its extracellular functions.^[^ ^44^, ^45^ ^]^ In addition, a previous study reported that in HCC cell lines, CXCL2 was mainly concentrated in the tumor supernatant rather than inside the tumor cells, which might have constrained the exploration of whether intracellular CXCL2 exerts distinct biological functions.^[^ ^19^ ^]^ These data may explain why CXCL2 exerts different biological effects in HCC. As we known, CXCL2 is transported via the classical secretory pathway (the endoplasmic reticulum‐Golgi pathway) and is released extracellularly after synthesis. Therefore, any factors that disrupt this pathway may lead to the intracellular accumulation of CXCL2. However, to the best of our knowledge, no study has specifically investigated the factors that promote CXCL2 retention within cells. Therefore, further detailed exploration of how to control intracellular CXCL2 expression is required.

Cholesterol metabolism plays a pivotal role in tumor progression and the regulation of the immune microenvironment.^[^ ^46^ ^]^ Multiple studies have reported that cholesterol and its metabolites can establish an immunosuppressive microenvironment and attenuate the antitumor effects of CD8^+^ T cells, macrophages, neutrophils, and so on.^[^ ^47^, ^48^ ^]^ In this study, we found that the addition of exogenous cholesterol or overexpression of SREBF2 reversed the polarization of neutrophils toward the antitumor phenotype induced by CXCL2 overexpression. The results of in vivo experiments also showed that CXCL2 overexpression mainly affected neutrophil polarization by depleting selective immune cells. There were no or only minimal alterations in the filtrating CD8⁺ T, CD4⁺ T, monocyte cell and macrophage populations in tumors overexpressing CXCL2, although there was an upward trend in the infiltration proportion and function of CD8⁺ T cells and macrophages. In this regard, we speculated that due to the significant increase in the number of tumor‐infiltrating neutrophils compared to other immune cells, neutrophils became the main cell type influenced by cholesterol under CXCL2 overexpression. Similarly, a recent study found that ZDHHC3 inhibition drastically enhanced CD4⁺ T cells infiltration in HCC. ZDHHC3 could promote cholesterol biosynthesis and mainly impair the cytotoxic function and number of CD4⁺ T cells with no or only minimal alterations in the infiltrating DC, B cell, and macrophage populations in tumors with Zdhhc3 knockdown.^[^ ^49^ ^]^ Liu et al. reported knockout of AURKB significantly inhibited CCA progression, reduced CD8^+^ T cell exhaustion and enhanced antitumor response by reducing cholesterol levels, because the number and function of CD8^+^ T cells were predominantly changed.^[^ ^50^ ^]^ These findings suggest that cholesterol exerts multiple effects on the functions of immune cells, which are intricately modulated by a plethora of factors under specific circumstances.

More importantly, our combination therapy of CXCL2 overexpression and anti‐PD‐1 reagent also demonstrated powerful therapeutic effects, with much more antitumor neutrophils and CD8^+^ T cells infiltration to retard HCC progression. These findings suggest that more animal and clinical experiments are needed to confirm the efficiency and stability after CXCL2 overexpression. Moreover, owing to the heterogeneity of the tumor microenvironment, the proposed combination strategies might only be effective in HCC with high CXCL2 expression, indicating the potential of CXCL2 as a predictive biomarker for patient stratification. Furthermore, as CXCL2 could modulate cholesterol synthesis, which in turn affects neutrophil function, therefore combining high CXCL2 expression with statin treatment may synergistically inhibit HCC progression. This promising combination strategy should be further investigated to improve the clinical translational potential of CXCL2‐based therapies.

## 4 Conclusion

In conclusion, our study highlighted the unconventional role of CXCL2 in repressing HCC through enhancing neutrophil‐mediated antitumor immunity. CXCL2 binds to YBX1 and impedes its nuclear translocation, resulting in decreased SREBF2 transcription and cholesterol biosynthesis. Concomitantly, it remodels the antitumor immune microenvironment, ultimately retarding the progression of HCC. These findings would contribute to our understanding of CXCL2 as a potential therapeutic target, and underscore the need for further research to fully elucidate its mechanisms and clinical applications.

## 5 Experimental Section

### Patients

Primary HCC and the adjacent non‐tumor tissues were collected from patients who underwent routine curative surgery at Henan Cancer Hospital (Zhengzhou, China). All HCC patients were confirmed by pathological diagnosis, and none of them received chemotherapy or radiotherapy before surgery. Eight normal liver tissues were obtained from the patients with hepatic hemangioma. The informed consent was obtained from each patient before the start of the study. This study was approved by the Ethics Committee of Peking University Health Science Center (IRB00001052‐12088).

### In Vivo Mice Experiments

C57BL/6J mice were purchased from the Department of Laboratory Animal Science, Peking University Health Science Center, and housed under specific pathogen‐free (SPF) conditions with a 12h light/dark cycle and free access to sterilized food and water. All procedures were approved by the Institutional Animal Care and Use Committee of Peking University Health Science Center (LA2021492).

For subcutaneous tumor model, Hepa 1–6 cells from control and CXCL2 overexpression groups were suspended in 100 µL PBS with 20% matrigel (Corning, USA) and subcutaneously injected into the right flank of mice. Tumor growth was monitored from day 7 post‐inoculation using digital calipers. Tumor volume was calculated as: Volume (mm^3^) = (Length × Width^2^) × 0.5. Mice were euthanized by cervical dislocation when the tumors reached 20 mm in diameter, followed by tumor excision, weighing, and processing for IHC or other analyses.

For experiments with immune cell depletion, mice were intraperitoneally injected with anti‐CD8α antibody (clone 2.43, Selleck, China, 200 µg per mouse before one day of inoculation and once every three days after inoculation) for CD8^+^ T cell depletion, clodrondate liposomes (Yeasen, China, 100 µL per mouse before 3 days of inoculation and once every four days after inoculation) for macrophage depletion and anti‐Ly6G antibody (clone 1A8, Selleck, China, 100 µg per mouse, thrice a week) for neutrophil depletion. For PD‐1 blockade therapy, anti‐PD‐1 antibody (clone RMP1‐14, Selleck, China, 100 µg per mouse, once every three days) treatment began when tumors reached ≈150 mm^3^.

For CCl_4_ combined diethylnitrosamine (DEN) induced HCC mouse model, 15‐day old C57BL/6J mice were injected intraperitoneally with DEN (2 mg kg^−1^, per mouse). Two weeks later, the mice were treated with CCl_4_ intraperitoneally at a dose of 5 mL kg^−1^ twice a week and liver tissues were collected from the 20th week after birth.

### Statistical Analysis

Data shown in all statistical graphs were presented as the mean ± standard deviation (SD), unless specifically indicated. Statistical analyses were carried out using SPSS software (version 22.0), GraphPad Software (version 8.0), or R software (version 4.0.0). Unless otherwise indicated, unpaired Student's *t* test or one‐way ANOVA analysis was applied for group comparisons. Two‐tailed *P* values less than 0.05 were considered as statistically significant.

### Ethics Approval and Consent to Participate

This study was approved by the Ethics Committee of Peking University Health Science Center. All research was conducted in accordance with both the Declarations of Helsinki and Istanbul. The informed consent was obtained from each patient prior to the start of the study (IRB00001052‐12088). All animal experimental procedures were approved by the Institutional Animal Care and Use Committee of Peking University Health Science Center (LA2021492).

## Conflict of Interest

The authors declare no conflict of interest.

## Author Contributions

X.L. and D.Y. contributed equally to this work. X.C., F.L., and L.W. conceived and designed the study; X.L., D.Y., Q.J., M.H., Z.Z., Y.L., Y.W., J.W., and T.Z. performed cell and animal experiments; X.L. and D.Y. analyzed the data; X.L., X.C., F.L., L.W., and G.W drafted the manuscript. All authors reviewed and approved the manuscript.

## Supporting information



Supporting Information

Supporting Information

## Data Availability

The data that support the findings of this study are available from the corresponding author upon reasonable request.
