# Supplementary material for: Tumor Cell‐Derived CXCL2 Potentiates Neutrophil‐Mediated Antitumor Immunity by Inhibiting Cholesterol Biosynthesis in Hepatocellular Carcinoma
Source: Adv Sci (Weinh). 2025 Oct 20;13(2):e11436. doi: 10.1002/advs.202511436 (PMC12786370; doi:10.1002/advs.202511436)
Supplement: Supplementary file 1 — Supporting Information [file ADVS-13-e11436-s001.docx]

Supporting Information

**Tumor Cell-Derived CXCL2 Potentiates Neutrophil-Mediated Antitumor Immunity by Inhibiting Cholesterol Biosynthesis in Hepatocellular Carcinoma**

Xin Liu; Danli Yang; Qianqian Jiang; Meng Han; Zhao Zhou; Yukun Li; Yu Wu; Jingzhou Wang; Ting Zhang; Guohua Lou; Guochao Wei; Lin Wang^*^; Fengmin Lu^*^; Xiangmei Chen^*^

### Materials and Methods

### Array-based comparative genomic hybridization (aCGH) and RNA expression profiles

The array-based [comparative genomic hybridization](https://www.sciencedirect.com/topics/immunology-and-microbiology/comparative-genomic-hybridization) (aCGH) assay of 25 paired HCC tissues and adjacent non-tumor tissues has been described in our previous study.^[1]^ The RNA expression profile in 6 pairs among the above 25 paired specimens was also described previously.^[2]^

### Data collection and processing

TCGA-LIHC dataset was obtained from the GDC website (https://portal.gdc.cancer.gov) and corresponding clinical information from UCSC xena website (https://xena.ucsc.edu/). HCC bulk RNA-seq datasets (GSE102083, GSE14520, GSE25097, GSE36376 and GSE62232) were downloaded from Gene Expression Omnibus (GEO) database and analyzed using R software. Raw expression data was normalized, and differential expression analysis was performed using the limma package. Pathway enrichment analysis was conducted with R package-ClusterProfiler.^[3]^ The relative score of 24 immune cells infiltration was evaluated by ssGSEA analysis. Moreover, ssGSEA analysis was also conducted to calculate the relative score of immune cells function based on corresponding genesets downloaded from MSigDB database (https://www.gsea-msigdb.org/gsea/msigdb/).

The expression status of CXCL2 in HCC and non-HCC tissues in 92 dataset was obtained from the Integrative HCC Gene Analysis (IHGA) database (https://www.hccdatasph.cn/app/ihga).^[4]^

HCC single-cell RNA-seq dataset (GSE242889) was obtained from GEO database and the HCC spatial RNA-seq dataset was downloaded from RRA database (https://data.mendeley.com/datasets/skrx2fz79n/1).^[5]^ Both were analyzed with standard procedures. The relative score of neutrophils function was calculated by AddModulescore and ssGSEA analysis. The genesets of neutrophils function were exhibited in **Table S3**.

The prognosis of immunotherapy data was obtained from Kapian-Meier Plotter (http://kmplot.com/analysis/) online tool and Tumor Immune Dysfunction and Exclusion (TIDE) database (http://tide.dfci.harvard.edu/).^[6]^

### Immunohistochemistry (IHC) and multi-color immunohistochemistry (mIHC)

The protocol of IHC was performed according to our previous study.^[7]^ The staining intensity and extent of CXCL2 protein were independently evaluated by two pathologists blinded to clinical data. Intensity was scored as 1 (negative), 2+ (weak), 3+ (moderate), or 4+ (strong). The percentage of positive tumor cells was categorized as 1 (< 25%), 3 (26-50%), 4 (51-75%), or 4 (>75%). A final immunoreactivity score (IRS) was calculated by multiplying intensity and percentage scores (range: 1-16). Cases with IRS > 8 were defined as high expression and IRS ≤ 8 as low expression.

For multi-color immunohistochemistry (mIHC), following the standard IHC procedures, the tissue sections were incubated with primary antibodies overnight at 4℃. Subsequently, they were incubated with secondary antibody and fluorescence staining diluted 1:100 in 1 × Plus Amplification Diluent. Once a staining cycle was completed, antigen retrieval was performed again to prepare for the next round of antibody staining. Finally, DAPI staining was carried out to label the cell nuclei and the slides were imaged. The information of antibodies was exhibited in **Table S4.**

### Cell lines

Human HCC cell lines (HCCLM3) and murine hepatoma cells (Hepa 1-6) were purchased from Shanghai Cell Bank, Chinese Academy of Sciences. HuH-1 and HEK 293T cell lines were purchased from the American Type Culture Collection (ATCC, Rockville, USA). All of them were maintained in Dulbecco’s Modified Eagle Medium (DMEM, Corning, USA) supplemented with 10% fetal bovine serum (FBS) (Sigma, USA), 100 U/mL penicillin, and 100 µg/mL streptomycin (Gibco, USA). Cells were cultured at 37°C in a humidified atmosphere containing 5% CO₂ and they were routinely tested to ensure mycoplasma negativity following standard procedures.

### Plasmid construction

Plasmids pLEX-MCS, pcDNA3.1, pGL3-Basic, Actin-Renilla were previously maintained and constructed in our own laboratory. The overexpression plasmid of human or mouse gene CXCL2 was constructed by inserting CXCL2 cDNA into the pLEX-MCS plasmid with a 3×Flag tag in C-terminal. The ΔCXCL2 overexpressing plasmid (signal peptide deletion of CXCL2) was also constructed accordingly. The overexpression plasmid of human gene YBX1 was constructed by inserting YBX1 cDNA into the pcDNA3.1 plasmid with a HA tag in N-terminal and the truncated mutants of YBX1 was constructed based on the full length of YBX1. The pLV3-CMV-SREBF2 (human)-CopGFP-Neo (P55062) plasmid was obtained from MiaoLingBio, China. The pGL3-Basic-SREBF2-promoter plasmid was constructed by cloning SREBF2-promoter into the pGL3-Basic plasmids. All plasmid transfections in cells were performed using Lipofectamine 2000 (Invitrogen, California, USA).

### Stable cell line construction and proliferative assays

2×10^5^ HCC cells were seeded in 6-well plate overnight, and lentivirus against human or mouse CXCL2/ΔCXCL2 according to the multiplicity of infection were added and incubated for 48 h. Then the cells were selected by puromycin (2μg/mL) for 7 days and the efficiency was validated by western blot.

For CCK-8 assay, cells were seeded in 96 well plates at a density of 3,000 cells per well and incubated for 1, 2, 3 and 4 days, respectively. Then CCK-8 solution was added and incubated for 1 hour before measuring absorbance at 450 nm.

For the colony formation assay, 500/1000 cells per well were seeded in 12/6-well plates and cultured for 1-2 weeks with medium changed every 2-3 days. Then they were fixed with 4% paraformaldehyde and stained with 0.1% crystal violet. Finally, the number of colonies was counted.

**Total RNA extraction, reverse transcription quantitative PCR (RT-qPCR) and RNA-seq analysis**

Total RNA was extracted from cells using TRIzol reagent following the manufacturer's protocol. cDNA was synthesized using HiScript III 1st Strand cDNA Synthesis kit (Vazyme, China), followed by qPCR using Roche Lightcycler480 II and SYBR green system (Genestar, China). The primers were shown in **Table S5**. The data were analyzed to determine the relative expression levels of target genes by 2^-ΔΔCT^.

RNA-seq was performed based on standard procedures. The sequencing data were analyzed using bioinformatics tools to identify differentially expressed genes, perform gene ontology (GO) enrichment analysis and gene set enrichment analysis (GSEA), then explore relevant signaling pathways between the control and CXCL2 overexpression HCCLM3 cells.

### Filipin III staining and cholesterol measurement

For Filipin III staining, cells were fixed with 4% paraformaldehyde for 15-20 minutes at room temperature and stained with 50 μg /mL filipin III for 30 min at 4 °C. Then images were photographed.

For cholesterol measurement, the total cholesterol assay Kit (E1015, Applygen, China) was used to quantify cholesterol levels according to the manufacturers.

### Flow Cytometry (FCM) analysis

The tumor tissues from subcutaneous tumor models were dissected and minced into approximate 1mm^3^ pieces. Then, they were enzymatically digested using collagenase D (Roche, Switzerland) and DNase I (Yeasen, China) at 37°C with gentle agitation for 1-2h to obtain single-cell suspensions. The immune cells were enriched using 40% percoll according to standard procedures. Subsequently, the cells were stained with relevant antibodies in the dark at 4°C for 30 minutes. After washing, the cells were analyzed using a cytoFlex Flow Cytometer (Beckman Coulter, USA) and processed with FlowJo software. The information of antibodies was exhibited in **Table S4.**

### Neutrophil extraction and in vitro co-culture experiments

The neutrophils from healthy donors were extracted according to standard protocols using Histopaque-1119 and Histopaque-1077 (sigma, USA). Then they were co-cultured with supernatants form HCC cells. After 12 hour’s co-culture, neutrophils were collected and washed with PBS. For apoptosis analysis by FCM, cells were stained with Annexin V-FITC and PI. For functional analysis, antibodies against iNOS and CD206 were used. The stained cells were analyzed by cytoFlex Flow Cytometer (Beckman Coulter, USA) and data was processed with FlowJo to assess apoptosis and function. The information of antibodies was exhibited in **Table S4.**

### Co-immunoprecipitation (Co-IP) assay

Co-IP experiments were carried out following the standard protocols. Briefly, cells were lysed using Cell lysis buffer for Western and IP (Beyotime, China) and incubated with relative antibodies overnight at 4℃ The next day, the protein A/G beads was added and incubated at 4℃ for 4 hours. The protein was detected by western blot or LC-MS/MS analysis. The information of antibodies was exhibited in **Table S4.**

### Nuclear-cytoplasmic protein fractionation

Nuclear and cytoplasmic proteins were isolated using the Nuclear and Cytoplasmic Protein Extraction Kit (Beyotime, China). Proteins were analyzed by western blotting to assess the localization and expression of YBX1.

### Western blot

The procedures of western blot were conducted as described previously.^[8]^ The information of antibodies was exhibited in **Table S4.**

### Liquid chromatography-tandem mass spectrometry (LC-MS/MS)

LC-MS/MS experiment was conducted as previously described.^[9]^ In brief, proteins obtained from co-immunoprecipitation (Co-IP) of HCCLM3 cells were lysed and dissolved in SDS buffer. Subsequently, they were stacked in SDS-PAGE gel, followed by coomassie blue staining. The entire protein band was then excised from the gel and chosen for further LC-MS/MS experiment by State Key Laboratory of Natural and Biomimetic Drugs, Peking University Health Science Center, Beijing.

### Confocal microscopy

The confocal microscopy was employed to visualize YBX1 localization and interaction with CXCL2. Cells were fixed, permeabilized, and stained with primary antibodies, followed by secondary antibodies conjugated with fluorophores. Then, cells were stained with DAPI (Beyotime, China) and observed using the TCS-SP8 STED 3X confocal microscope (Leica, Germany). The information of antibodies was exhibited in **Table S4**.

### Dual-Luciferase reporter assay

For determination of the effects of YBX1 on SREBF2 promoter activity, PGL3-derived luciferase reporter vectors bearing the SREBF2 promoter was co- transfected with Actin-Renilla luciferase vectors into HCC cells with CXCL2/ΔCXCL2 overexpression. Luciferase activity in cytoplasmic extracts was measured 48 h after transfection using Dual Luciferase Reporter Assay kit (Promega, USA). Firefly luciferase activity was normalized to Renilla luciferase activity in the same cell extract and presented as a ratio of firefly/Renilla luciferase activity.

### RNA stability and RIP experiments

To analyze RNA stability, HCC cells from control and CXCL2 overexpression groups were treated with actinomycin D at various time intervals (0, 3, 6, 9, 12 and 15 hours). Total RNA was extracted from samples and reversed HiScript III 1st Strand cDNA Synthesis kit (Vazyme, China). The RT-qPCR was used to measure the relative abundance of the target RNA at each time point.

The procedure of RIP experiment was performed as described before.^[9]^ The HCC cells from YBX1 overexpression group were cultured and lysed in a buffer with protease and RNase inhibitors. The rabbit anti-HA antibody and anti-IgG were incubated with protein A/G magnetic beads, which have been pre-cleared to reduce non-specific binding. Then the cell lysate was added overnight at 4℃. Finally, the RNA was isolated and analyzed by qPCR to identify and quantify the target RNAs interacting with YBX1.

### Chromatin Immunoprecipitation (ChIP) assay

ChIP assay was performed according to the manufacturer’s instructions and described in our previous study.^[7]^ Briefly, the cell lysis supernatants, which were cross-linked and sonicated, were incubated with anti-Rabbit HA, anti-Rabbit YBX1 or anti-Rabbit IgG antibody overnight at 4℃. The next day, sheared chromatin was mixed with protein A/G beads and incubated at 4℃ for 4 hours. The precipitated DNA was analyzed by RT-qPCR for the presence of SREBF2 binding sites. Primer sequences are listed in **Table S5**.

### References

[1] JIANG S, YANG Z, LI W, et al. Re-evaluation of the carcinogenic significance of hepatitis B virus integration in hepatocarcinogenesis[J]. PLoS One, 2012, 7:e40363. DOI: 10.1371/journal.pone.0040363.

[2] LI X, ZHANG J, YANG Z, et al. The function of targeted host genes determines the oncogenicity of HBV integration in hepatocellular carcinoma[J]. J Hepatol, 2014, 60:975-984. DOI: 10.1016/j.jhep.2013.12.014.

[3] YU G, WANG LG, HAN Y, et al. clusterProfiler: an R package for comparing biological themes among gene clusters[J]. Omics, 2012, 16:284-287. DOI: 10.1089/omi.2011.0118.

[4] ZHANG Q, HU W, XIONG L, et al. IHGA: An interactive web server for large-scale and comprehensive discovery of genes of interest in hepatocellular carcinoma[J]. Comput Struct Biotechnol J, 2023, 21:3987-3998. DOI: 10.1016/j.csbj.2023.08.003.

[5] LI K, ZHANG R, WEN F, et al. Single-cell dissection of the multicellular ecosystem and molecular features underlying microvascular invasion in HCC[J]. Hepatology, 2024, 79:1293-1309. DOI: 10.1097/hep.0000000000000673.

[6] KOVáCS SA, FEKETE JT, GYŐRFFY B. Predictive biomarkers of immunotherapy response with pharmacological applications in solid tumors[J]. Acta Pharmacol Sin, 2023, 44:1879-1889. DOI: 10.1038/s41401-023-01079-6.

[7] ZHANG T, GUAN G, ZHANG J, et al. E2F1-mediated AUF1 upregulation promotes HCC development and enhances drug resistance via stabilization of AKR1B10[J]. Cancer Sci, 2022, 113:1154-1167. DOI: 10.1111/cas.15272.

[8] GU Z, JIANG Q, ABULAITI A, et al. Hepatitis B virus enhancer 1 activates preS1 and preS2 promoters of integrated HBV DNA impairing HBsAg secretion[J]. JHEP Rep, 2024, 6:101144. DOI: 10.1016/j.jhepr.2024.101144.

[9] ZHANG T, ZHENG H, LU D, et al. RNA binding protein TIAR modulates HBV replication by tipping the balance of pgRNA translation[J]. Signal Transduct Target Ther, 2023, 8:346. DOI: 10.1038/s41392-023-01573-7.


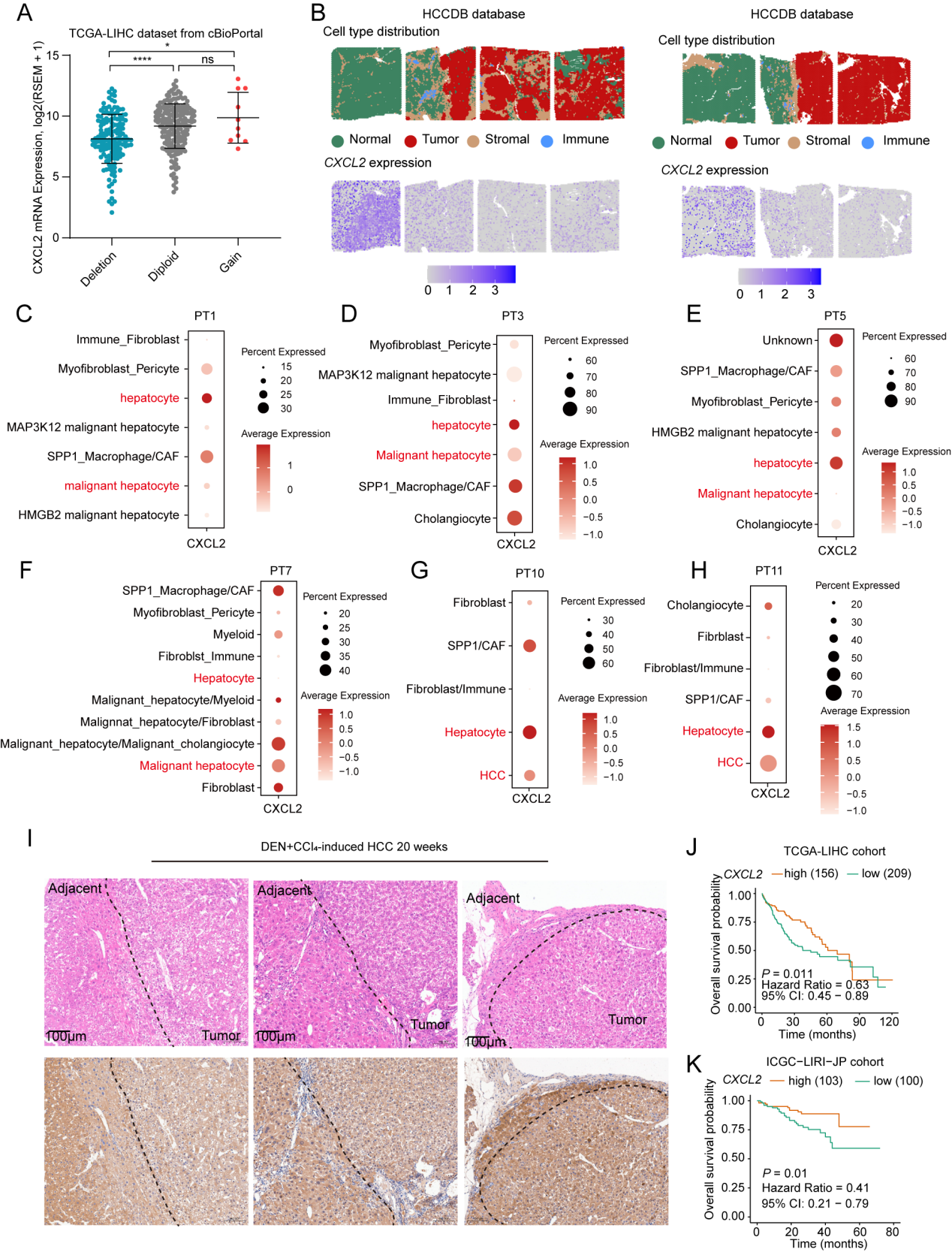


**Figure S1. CXCL2 was downexpressed in HCC tissues.** (A) The CNVs analysis of CXCL2 in HCC tissues in TCGA-LIHC dataset from cBioportal. (B) The spatial expression feature of CXCL2 in HCC from HCCDB database. (C-H) The expression feature of CXCL2 mRNA from HCC spatial transcriptomics dataset obtained from Mendeley database. (C) PT1; (D) PT3; (E) PT5; (F) PT7; (G) PT10; (H) PT11. (I) The HE and IHC staining image of spontaneous HCC mouse models using CXCL2 antibody. (J, K) Kaplan-Meier analysis of survival of patients with HCC classified as CXCL2 expression from TCGA-LIHC (J), ICGC-LIRI-JP (K) database. Data were presented as mean ± SD. * *P* < 0.05, **** *P* < 0.0001, ns, no significance. *P* value was calculated by one-way ANOVA (A) and log rank test (J, K).


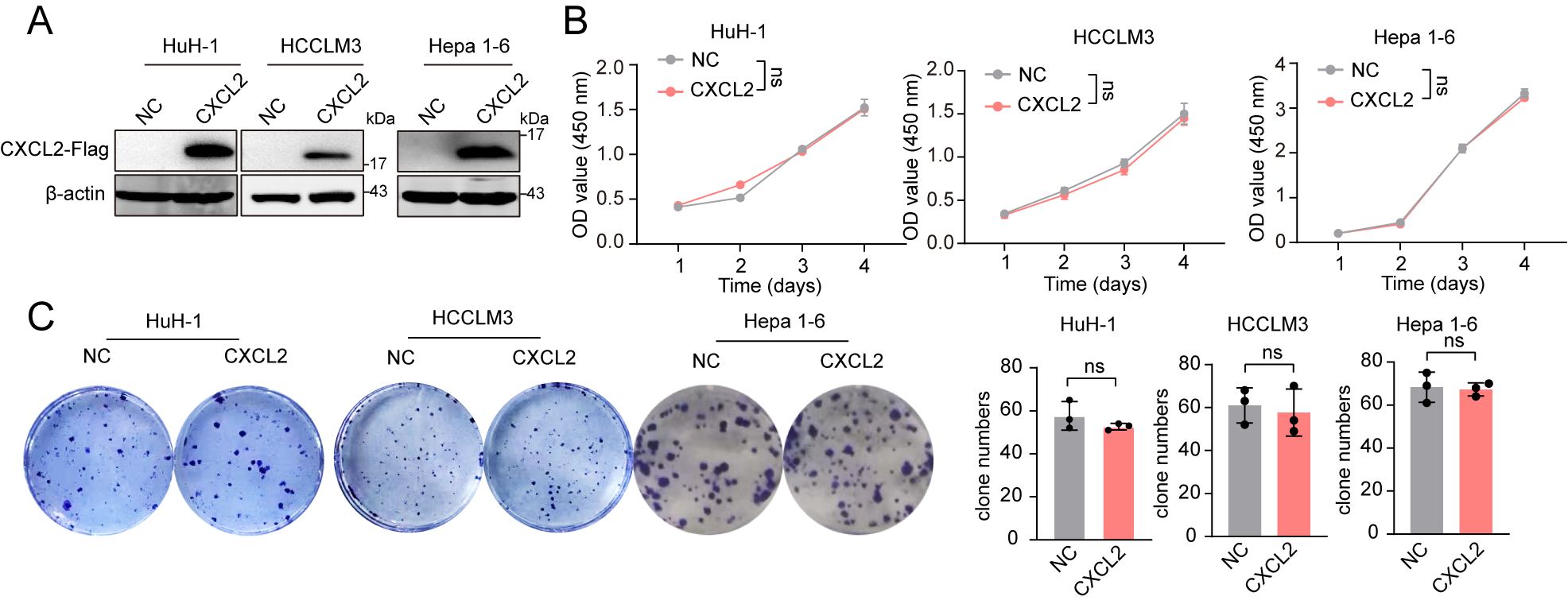


**Figure S2. CXCL2 overexpression had no significant impact on the proliferation capacity of HCC cells.** (A) The identification of stably CXCL2 overexpressed HCC cells by western blot. (B, C) The proliferation of HCC cells with negative control (NC) or CXCL2 overexpression was evaluated by CCK-8 analysis (B) (n=4) and colony formation (C) (n=3). Data were all presented as mean ± SD. ns, no significance. *P* value was calculated by two-way ANOVA (B) and Student’s *t* test (C).


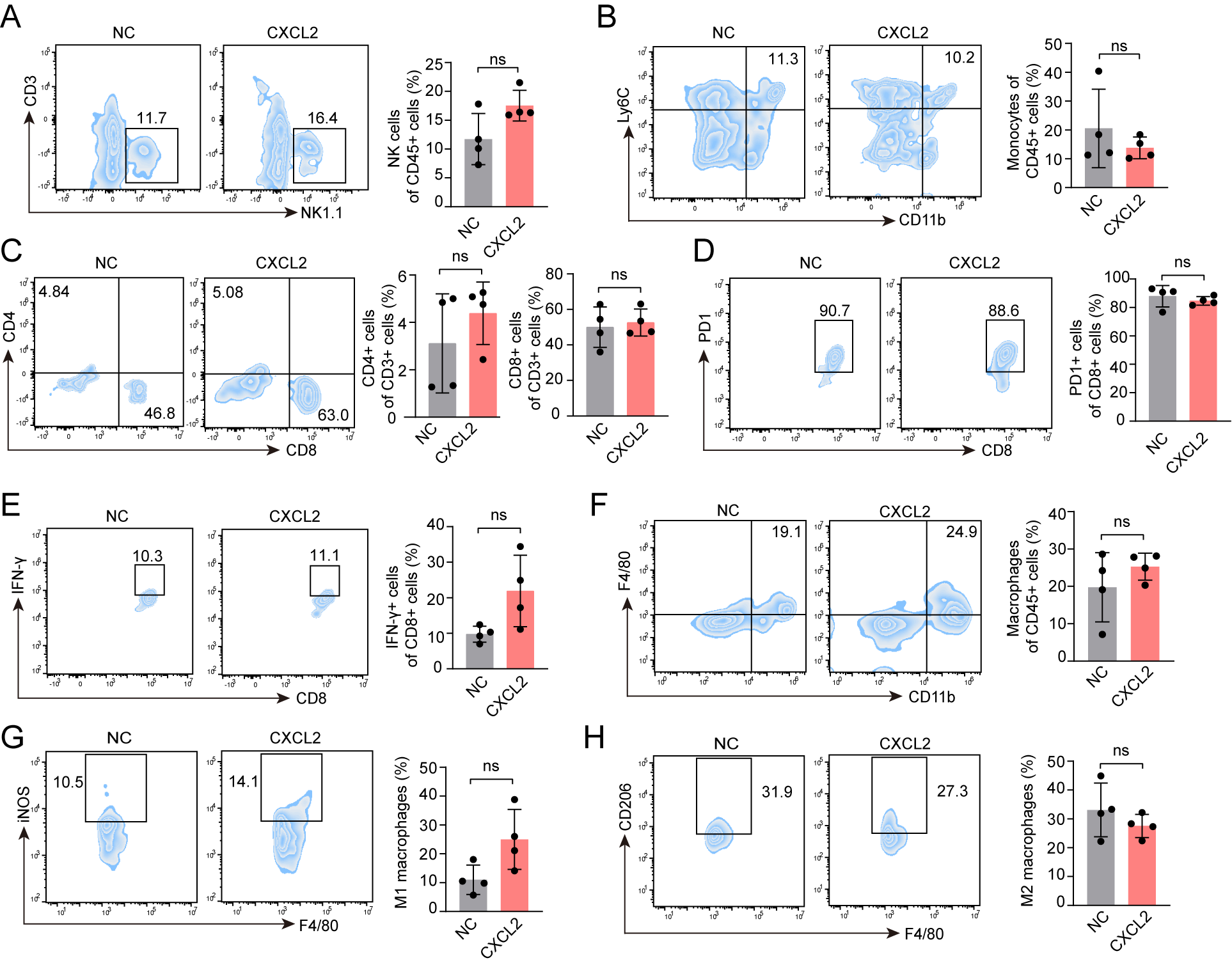


**Figure S3. The FCM results of various types of immune cells in NC and CXCL2 overexpressing subcutaneous tumors.** (A-H) The FCM analysis of the proportion of tumor-infiltrating NK cells (A), monocytes (B), CD4^+^/CD8^+^ T cells (C), PD1^+^ CD8^+^ T cells (D), IFNγ^+^ CD8^+^ T cells (E), macrophages (F), M1 macrophages (G) and M2 macrophages (H) (n=4). Data were all presented as mean ± SD. ns, no significance. *P* value was calculated by Student’s *t* test.


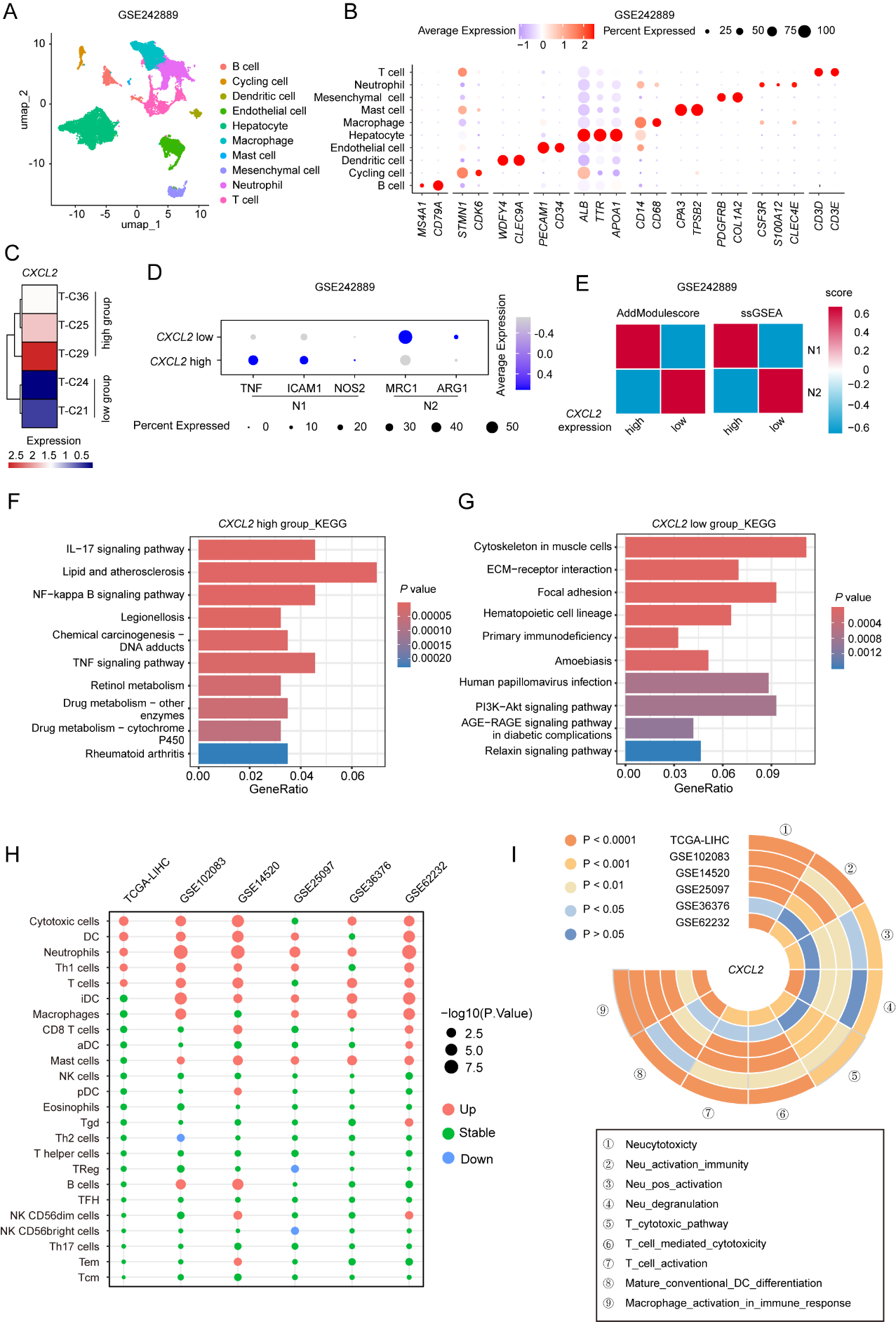


**Figure S4. CXCL2 was positively associated with neutrophils polarization towards anti-tumor phenotype through bioinformatics analysis.** (A) The UMAP plot of the ten cell clusters (B cells, cycling cells, dendritic cells, endothelial cells, hepatocyte, macrophage, mast cells, mesenchymal cells, neutrophil and T cells) from five HCC samples in GSE242889 dataset. (B) Heatmap of the specific cell markers for the ten cell clusters. (C) According to the average expression of CXCL2 mRNA expression, HCC samples were divided into high (n=3) and low (n=2) groups. (D) Average expression of related genes signified as N1 or N2 neutrophil markers in CXCL2 high or CXCL2 low group, respectively. (E) The relative score of N1-like signature or N2-like signature in CXCL2 high or CXCL2 low group calculated by AddModulescore and ssGSEA analysis. (F, G) KEGG analysis of neutrophils in CXCL2 high group (F) or CXCL2 low group (G). (H) The correlation of CXCL2 mRNA expression with various kinds of immune cells in TCGA and GEO databases. (I) The comparison of 9 genesets of immune cell function between CXCL2 high group and CXCL2 low group in TCGA and GEO databases.


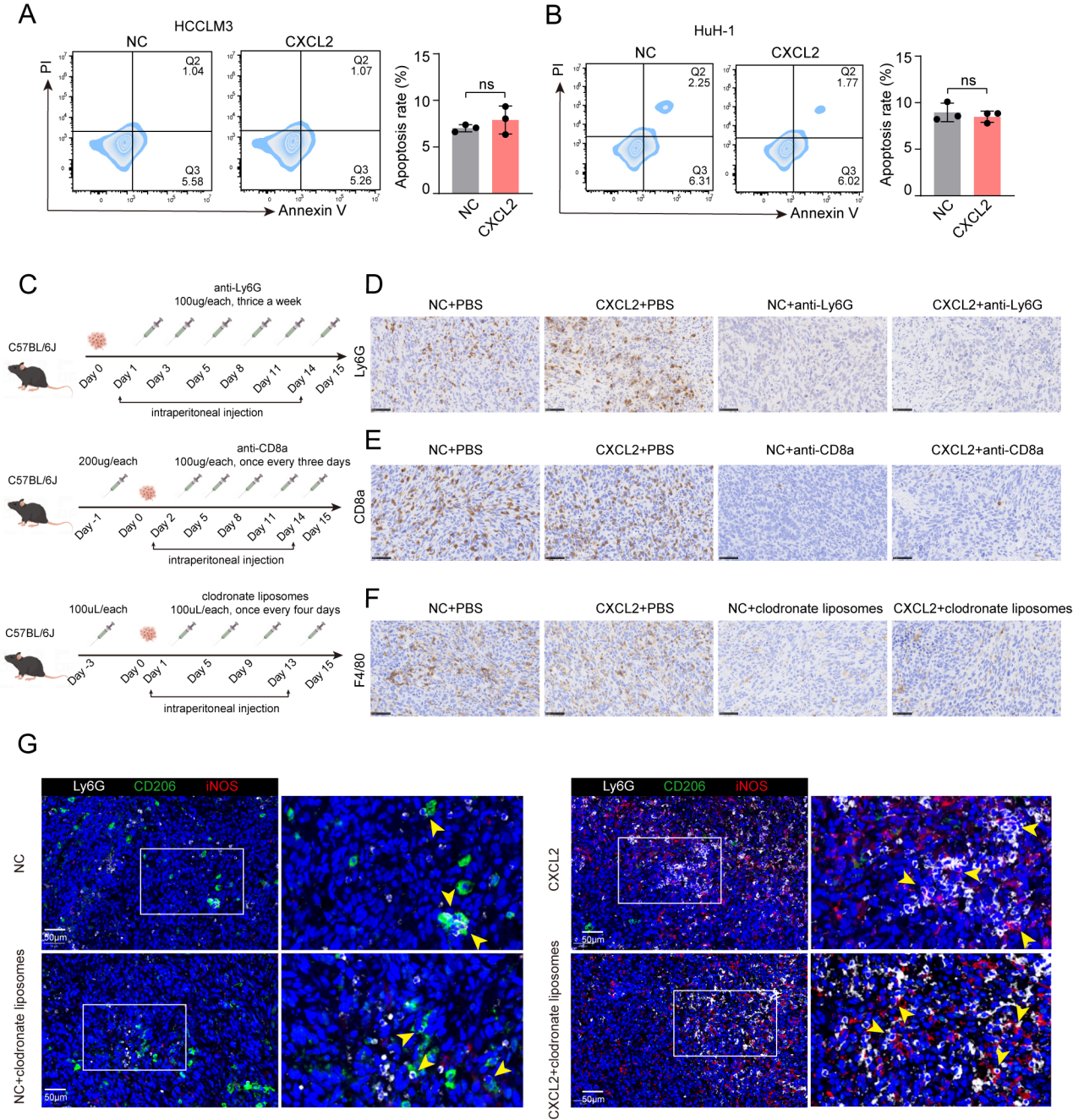


**Figure S5. The efficiency of immune cell depletion.** (A, B) The FCM analysis showed the apoptosis rate of neutrophils co-cultured with conditioned medium of NC and CXCL2 overexpression HCCLM3 (A) or HuH-1 (B) cells for 12h (n=3). (C) Treatment schedule of anti-Ly6G, anti-CD8a and clodronate liposomes in subcutaneous tumor in C57BL/6J mice inoculated with NC or CXCL2 overexpression Hepa 1-6 cells. (D-F) The IHC results showed the depletion efficiency of neutrophils (D), CD8^+^ T cells (E) and macrophages (F). (G) The representative image of multicolor immunohistochemical staining of Ly6G, CD206 and iNOS. Data were presented as mean ± SD. ns, no significance. *P* value was calculated by Student’s *t* test (A and B).

**
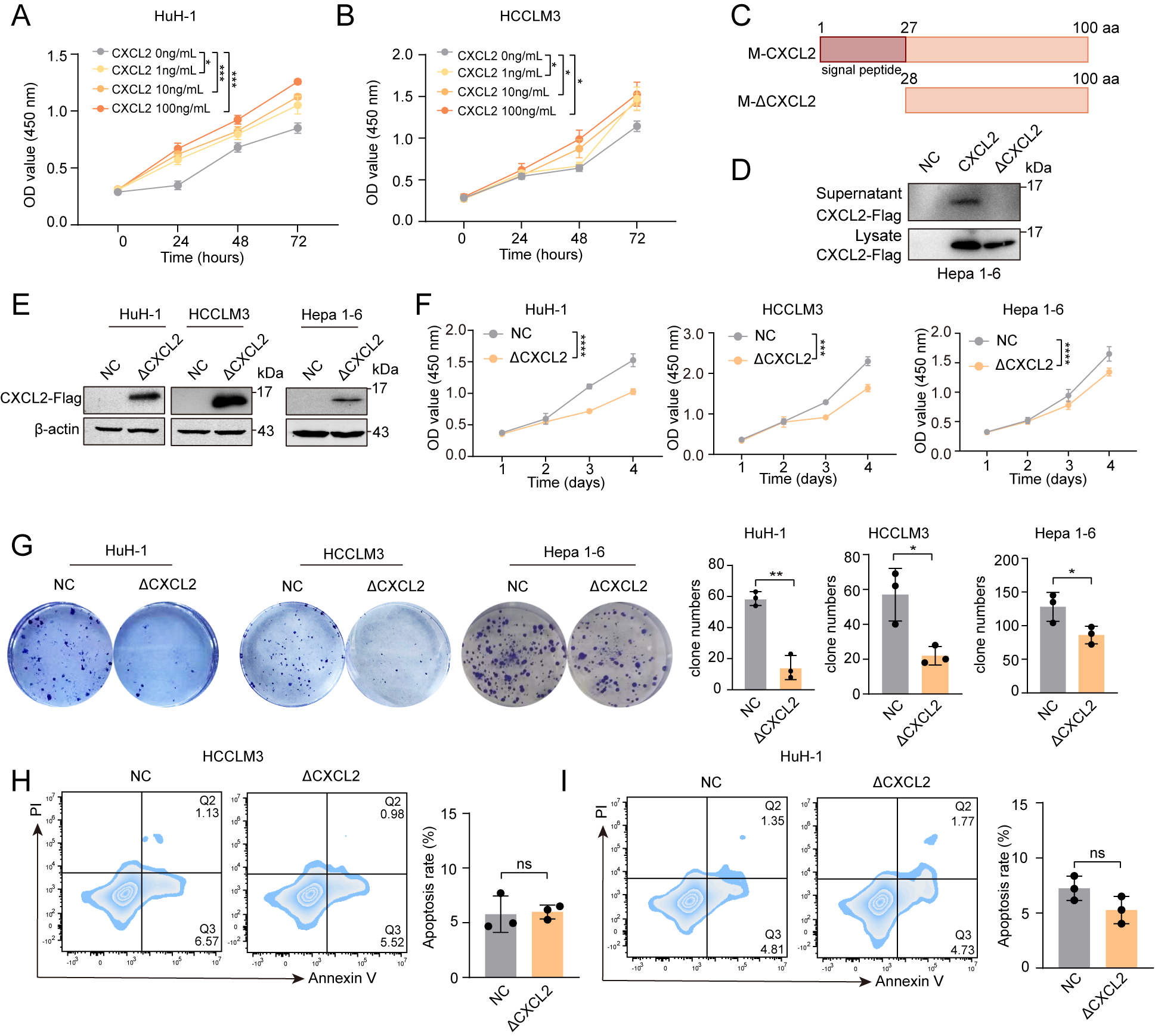
**

**Figure S6. Intracellular CXCL2 inhibited HCC proliferation.** (A, B) The proliferation of HuH-1 (A) and HCCLM3 (B) cells treated with different concentration of recombinant exogenous CXCL2 protein by CCK-8 analysis (n=4). (C) The schematic representation of the full-length CXCL2 and signal peptide deletion (ΔCXCL2). (D) The supernatants and cell lysates from Hepa 1-6 cells with Flag-tagged CXCL2 or ΔCXCL2 overexpressed were determined by western blot using anti-Flag antibody. (E) The identification of stably ΔCXCL2 overexpressed HCC cells by western blot. (F, G) The proliferation of HCC cells between NC and ΔCXCL2 overexpression by CCK-8 analysis (F) (n=4) and colony formation (G) (n=3). (H, I) The FCM analysis showed the apoptosis rate of neutrophils co-cultured with conditioned medium of control and ΔCXCL2 overexpression HCCLM3 (H) and HuH-1 (I) cells for 12h (n=3). Data were all presented as mean ± SD. * *P* < 0.05, ** *P* < 0.01, *** *P* < 0.001, **** *P* < 0.0001, ns, no significance. *P* value was calculated by two-way ANOVA (A, B and F) and Student’s *t* test (G, H and I).


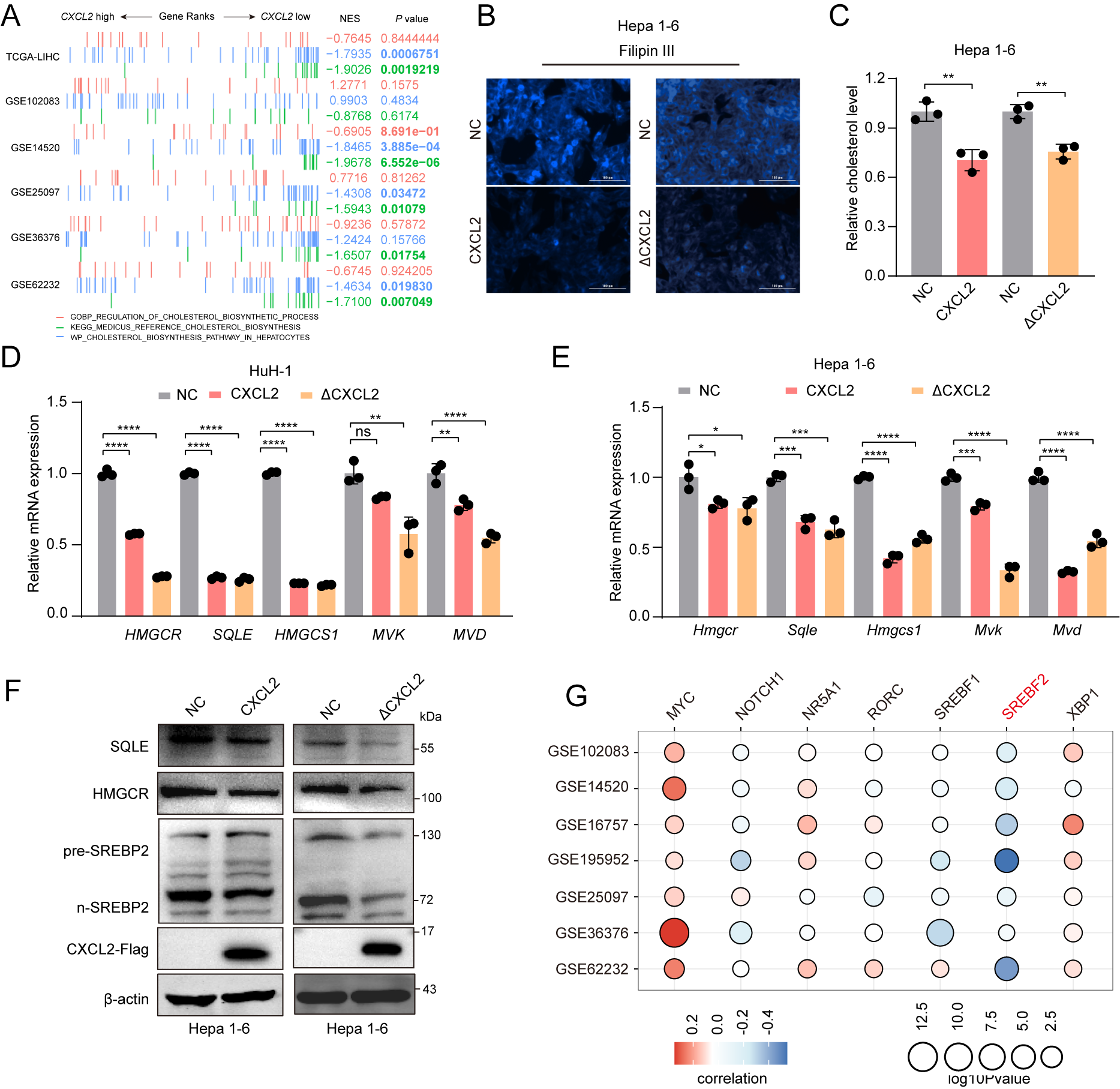


**Figure S7. CXCL2 suppressed cholesterol biosynthesis in HCC.** (A) GSEA analysis of cholesterol biosynthesis pathways based on CXCL2 mRNA expression from TCGA and GEO database. (B, C) The representative image of Filipin III staining of cholesterol (B) and the relative cholesterol measurement (C) (n=3) in NC and CXCL2/ΔCXCL2 overexpression Hepa 1-6 cells. (D, E) The relative mRNA expression of cholesterol biosynthesis related genes in NC and CXCL2/ΔCXCL2 overexpression HuH-1 (D) and Hepa 1-6 (E) cells (n=3). (F) The relative protein expression of cholesterol biosynthesis related genes in NC and CXCL2/ΔCXCL2 overexpression Hepa 1-6 cells were detected by western blot. (G) The correlation of key transcription factors for cholesterol biosynthesis with CXCL2 mRNA expression in 7 HCC datasets from GEO database. Data were all presented as mean ± SD. * *P* < 0.05, ** *P* < 0.01, *** *P* < 0.001, **** *P* < 0.0001, ns, no significance. *P* value was calculated by Student’s *t* test (C) and one-way ANOVA (D and E).


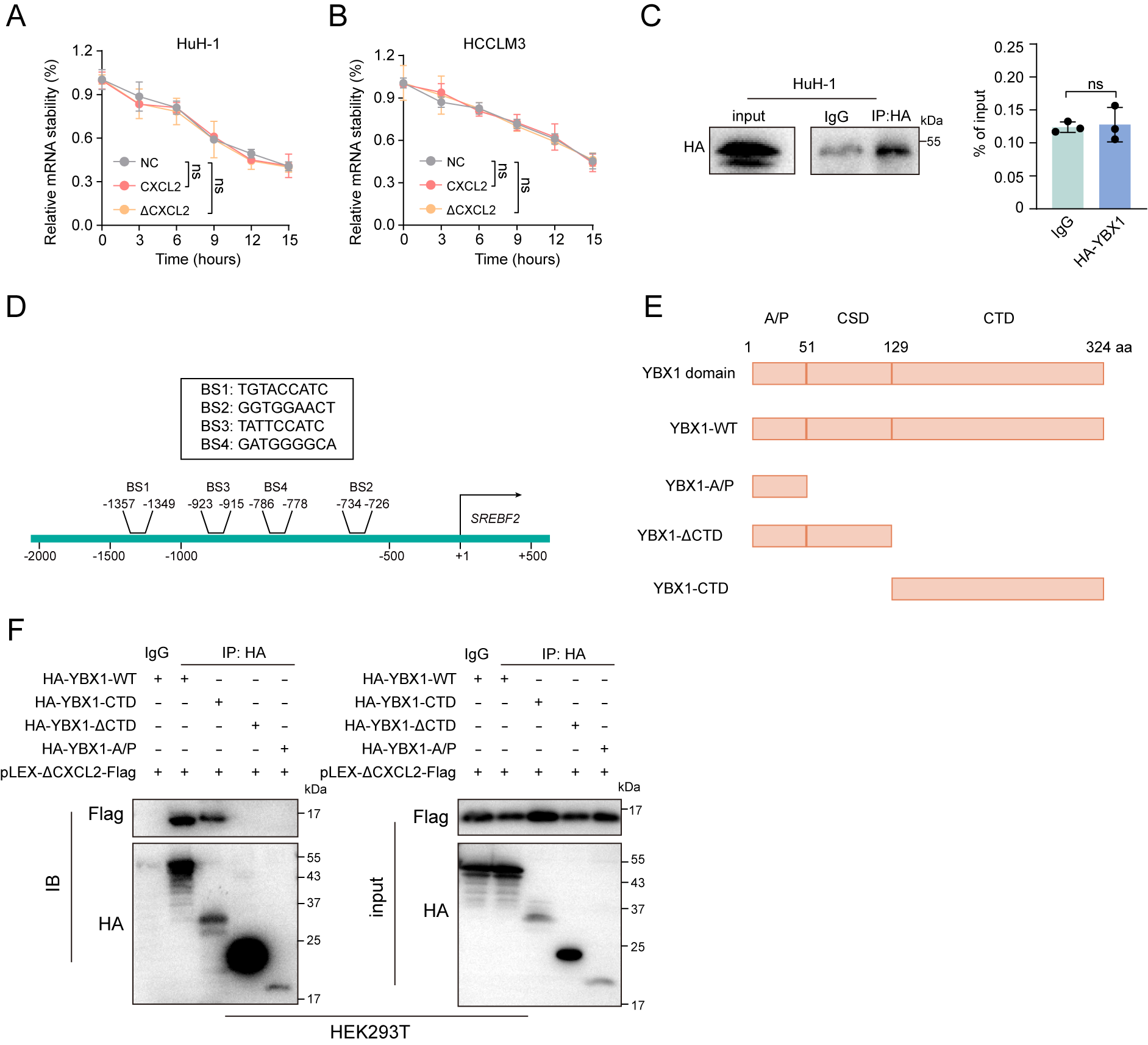


**Figure S8. CXCL2 reduced SREBF2 transcription by interacting with YBX1.** (A, B) The mRNA stability of SREBF2 in NC and CXCL2/ΔCXCL2 overexpression in HuH-1 (A) or HCCLM3 (B) cells (n=3). (C) The RIP analysis showed YBX1 was unable to bind to SREBF2 mRNA (n=3). (D) The four putative binding sites of YBX1 on SREBF2 mRNA predicted by JASPAR database. (E) Schematic representation of the full length and three truncated mutants of YBX1. (F) HEK293T cells was co-transfected with pLEX-ΔCXCL2-Flag plasmid with YBX1 truncated mutants for 48h. Co-IP assay was performed using IgG or anti-HA antibody. Data were all presented as mean ± SD. ns, no significance. *P* value was calculated by two-way ANOVA (A and B) and Student’s *t* test (C).


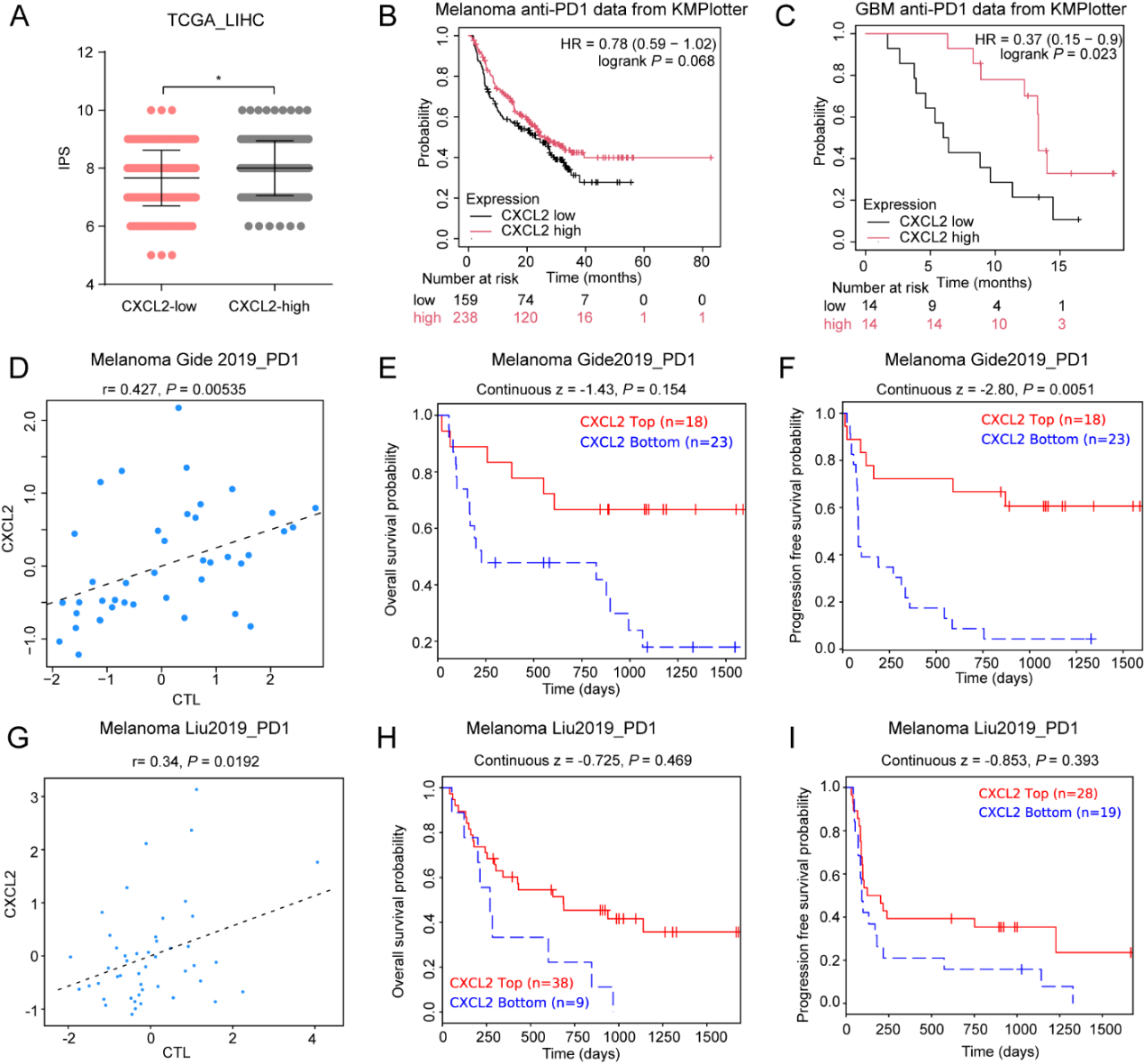


**Figure S9. The high CXCL2 expression was correlated with enhanced sensitivity to anti-PD1 immunotherapy in melanoma and glioblastoma (GBM) patients.** (A) The IPS score of CXCL2 low and CXCL2 high group in HCC patients from TCGA-LIHC dataset. (B, C) Survival analysis of patients with melanoma (B) or GBM (C) treated with anti-PD1 based on CXCL2 mRNA expression from KMPlotter database. (D, G) The correlation of CXCL2 mRNA expression with abundance of cytotoxic T cells (CTL) in tumor tissues from TIDE database. (E, H) Survival analysis of overall survival in patients with melanoma with anti-PD1 based on CXCL2 mRNA expression from TIDE database. (F, I) Survival analysis of progression free survival (PFS) in patients with melanoma with anti-PD1 based on CXCL2 mRNA expression from TIDE database. Data are presented as the mean ± SD. * *P* < 0.05. *P* value was calculated by Student’s *t* test (A).
